# Climate stress resistance in male Queensland fruit fly varies among populations of diverse geographic origins and changes during domestication

**DOI:** 10.1186/s12863-020-00935-2

**Published:** 2020-12-18

**Authors:** Ángel-David Popa-Báez, Siu Fai Lee, Heng Lin Yeap, Shirleen S. Prasad, Michele Schiffer, Roslyn G. Mourant, Cynthia Castro-Vargas, Owain R. Edwards, Phillip W. Taylor, John G. Oakeshott

**Affiliations:** 1 Applied BioSciences, Macquarie University, Sydney, NSW 2109 Australia; 2 Land and Water, CSIRO, Canberra, ACT 2601 Australia; 3 Daintree Rainforest Observatory, James Cook University, Cape Tribulation, QLD 4873 Australia

**Keywords:** *Bactrocera tryoni*, Heat resistance, Desiccation resistance, Ecotypic variation, Domestication effects

## Abstract

**Background:**

The highly polyphagous Queensland fruit fly (*Bactrocera tryoni* Froggatt) expanded its range substantially during the twentieth century and is now the most economically important insect pest of Australian horticulture, prompting intensive efforts to develop a Sterile Insect Technique (SIT) control program. Using a “common garden” approach, we have screened for natural genetic variation in key environmental fitness traits among populations from across the geographic range of this species and monitored changes in those traits induced during domestication.

**Results:**

Significant variation was detected between the populations for heat, desiccation and starvation resistance and wing length (as a measure of body size). Desiccation resistance was correlated with both starvation resistance and wing length. Bioassay data for three resampled populations indicate that much of the variation in desiccation resistance reflects persistent, inherited differences among the populations. No latitudinal cline was detected for any of the traits and only weak correlations were found with climatic variables for heat resistance and wing length. All three stress resistance phenotypes and wing length changed significantly in certain populations with ongoing domestication but there was also a strong population by domestication interaction effect for each trait.

**Conclusions:**

Ecotypic variation in heat, starvation and desiccation resistance was detected in Australian Qfly populations, and these stress resistances diminished rapidly during domestication. Our results indicate a need to select source populations for SIT strains which have relatively high climatic stress resistance and to minimise loss of that resistance during domestication.

## Background

Climate plays a major role in determining the geographical distribution of species, and their climate adaptability is considered a key component of their expansion and invasion potential [1, 2]. The Queensland fruit fly (*Bactrocera tryoni* Froggatt) (‘Qfly’) is native to subtropical regions of eastern Australia but has also established populations in the Northern Territory and down the east coast to temperate regions of Victoria, with some invasive populations also found in New Caledonia, French Polynesia, Pitcairn Island and Cook Island [3, 4]. As its range has expanded so has its pest status; it is now the major insect pest of Australian horticulture [5, 6]. The species’ wide climatic and geographical range suggests a high level of climate adaptability.

Latitudinal clines in climatic stress resistance and life history traits are well documented in insects, particularly in *Drosophila melanogaster* [7]*.* In some cases, such as body size variation in *Drosophila*, strong clinal signals are detected in different continents [8–12]. These parallel geographical patterns reflect repeatable adaptive genetic responses to climatic selection. In contrast, latitudinal patterns of starvation resistance in *D. melanogaster* differ among continents: a strong cline is present in India [13], but absent in South America [14] and Australia [15]. Furthermore, while linear clines are abundant in insects, non-linear patterns also exist (e.g., overwinter egg production and longevity in *D. melanogaster* [16]). In addition to these classical examples in *D. melanogaster*, clinal variations have also been observed in other insects such as *D. birchii* and *D. serrata* in eastern Australia [7, 17–19] and *D. subobscura*, *Eldana saccharina*, *Glossina pallidipes,* and *Anopheles gambiae* in other continents [20–23]. In several cases the phenotypic variation in traits such as climate stress resistance has been associated with gradients in specific climatic variables [17, 19–22, 24–28] and, in physiological terms, with differences in body size and sometimes also developmental rates [10, 12, 17, 18, 29, 30].

In Qfly, survival and reproduction are heavily influenced by temperature, moisture and availability of suitable host fruits [31–35], and Fletcher [36, 37] has suggested that the populations now persisting in the temperate regions do so in part by short distance movements between orchards and nearby water sources. Desiccation stress is therefore speculated to be a major determinant of Qfly distributions [32, 35, 38], although there is as yet no empirical data in the literature about variation in desiccation resistance amongst wild populations of the species. Not much is known about variation in Qfly thermal resistance either. Bateman [39] reported that northern populations of Qfly have higher fecundity and survival rates at higher temperatures (30 °C) than at the standard laboratory rearing conditions of 25 °C. However, he acknowledged some possible bias in his work through the inclusion of what is now recognised as a sibling species, *B. neohumeralis* (which he termed *‘variety neohumeralis’*), in some of his collections. Furthermore, Meats [32] found no difference in cold resistance between northern and southern Qfly collections and he later reported rapid acclimation to low temperatures in several populations in the laboratory. Overall the current literature on geographic variation in the climatic stress resistances of Qfly is fragmentary and provides insufficient detail for understanding the role of such variation in the ecology and invasive potential of the species, and some of the literature is not based on current taxonomy [6, 38].

Better understanding of genetic variation in Qfly climate stress resistance has direct relevance to the Sterile Insect Technique (SIT) programmes now being implemented to suppress established populations and eliminate new outbreaks in key horticultural regions. These bisexual SIT programmes involve the mass rearing, gamma ray sterilisation and mass release of a domesticated Qfly strain into outbreak areas, resulting in wild flies wasting their reproductive effort on non-productive mating. These programmes are now being carried out in several regions of south-eastern Australia [40]. Climate stress resistances (along with other life history traits outside the scope of this paper such as mating competitiveness [41–43], lifespan [44] and predator evasion [45]) have been identified as important for the success of these programmes [46–48]. Some climate stress resistance is lost during the domestication process that occurs when the large numbers of Qflies that need to be produced for irradiation and release are mass reared in factory settings for multiple generations [46, 49, 50]. Changes in various traits during domestication have also been observed in strains of Mexican fruit fly *Anastrepha ludens* used in SIT programmes [51]. Given the harsh conditions into which the flies are often released, it is important to select strains for mass rearing from relatively stress resistant populations and to maintain as much of that resistance through multiple generations of mass rearing as possible.

The present study adopts a “common garden” approach to investigate genetic variation in heat, cold, desiccation and starvation resistance in Qfly populations of diverse geographical origins. We also explore how the variation in those traits relates to wing length (as a measure of body size), geographic origin (latitude and coastal vs inland) and weather variables. Additionally, we investigate how the climate stress resistances change during domestication and whether those changes differ between populations. Populations from three sites showing desiccation resistance differences in the initial survey were resampled in subsequent years and rescored for that trait to determine the genetic stability of the population differences observed. We discuss our results in relation to the ecology of this invasive pest and implications for SIT-based pest control programmes.

## Results

### Heat resistance

**Fig. 1:**
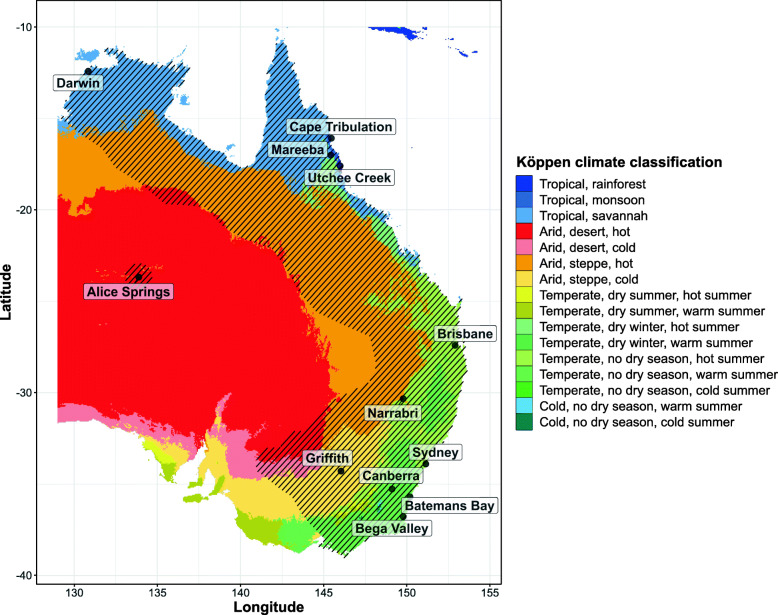
Geographical distribution of Qfly populations studied. Climatic zones are as described in the Köppen climate classification scheme for the years 1980 to 2016 as updated by Beck et al. [85]. Hatched areas encompass the known distribution of Qfly across Australia. Note that Qfly populations in the arid inland regions of Australia are largely confined to townships and irrigated areas. The map was created with *‘ggplot2’* [109] in R using climate zones data from Beck et al. [85] under the Creative Commons Attribution 4.0 International Licence

**Fig. 2:**
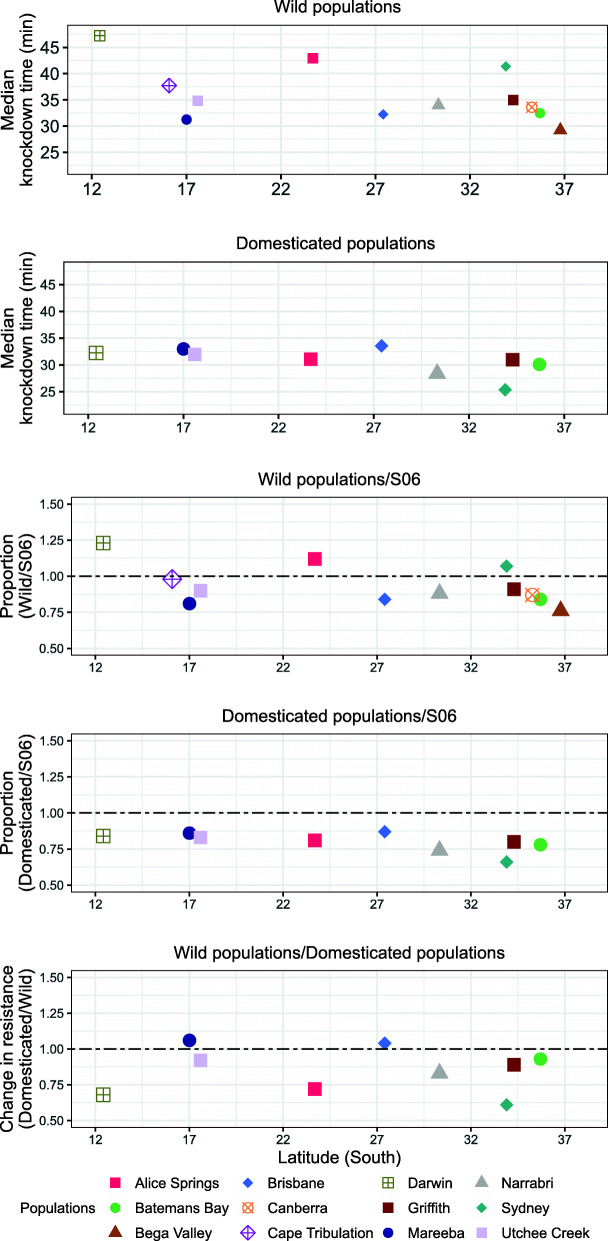
Variation in heat resistance of wild and domesticated Qfly populations. Data are first presented as normalised median knockdown times in minutes on exposure to 42 °C for each population in the generations designated as wild (G2/G3) and domesticated (G11–15). The data for both the wild and domesticated generations are then also shown as ratios against the S06 control used in the same batch of bioassays. Finally, the ratios of the median knockdown times for the domesticated vs wild generations for each population are given. Standard errors for the knockdown times were on average 5.34 and 2.55 for the wild and domesticated populations respectively. Note that not all populations were rescored at G11–15

Our primary survey of 12 populations from widely separated sites (Fig. 1**,** Table 1**,** Additional file 1 Table S1) found significant population differences at G2/G3 (${\chi}_{11}^2$ = 5.17, dispersion (ϕ) = 0.098, *P* < 0.001) in heat resistances as measured by knockdown time at 42 °C (Fig. 2**)**. These differences were not explained by latitude (*t*_8_ *=* 1.72*, P* > 0.05), or coastal vs inland origin of the populations (*t*_8_ = 0.84, *P* > 0.05). There was a significant association between the differences in heat resistance and the geographical distance between populations (Mantel’s r = 0.46, *P =* 0.02), although it only explained 21% of the variance in resistance. The resistance differences were largely due to the relatively long knockdown times (i.e., higher resistances) of the Darwin, Alice Springs and Sydney populations (on average, 32% longer than those of the other populations).

**Table 1:** Populations studied in the primary and resampled surveys

Population	Approximate location		Origin	Source fruits	Time of year (Season)	Year of collection	Bioassayed generation	
	Latitude	Longitude					Heat and Cold	Desiccation and Starvation
Darwin	−12.42	130.85	Coastal	Carambola/Stone fruits	Nov (Wet)	2016	G2/G15	G2
Cape Tribulation	−16.09	145.46	Coastal	Carambola	Mar (Wet)	2017	G2	G2
Mareeba	−17.01	145.43	Inland	Carambola/Guava	Feb (Wet)	2017	G3/G13	G2, G4, G6, G8, G11
Utchee Creek	−17.60	145.99	Coastal	Carambola	Mar (Wet)	2017	G2/G12	G2, G4, G6, G8, G10
Alice Springs	−23.69	133.89	Inland	Stone fruits	Nov (Spring)	2016	G2/G15	G2, G4, G6, G8, G10
Brisbane	−27.41	152.90	Coastal	Guava	Mar (Wet)	2017	G2/G12	G2, G4, G6, G8, G10
Narrabri	−30.33	149.78	Inland	Guava/Stone fruits	Mar (Wet)	2017	G2/G12	G2, G4, G6, G8, G10
Sydney	−33.90	151.14	Coastal	Loquats/Mulberries	Nov (Spring)	2016	G2/G15	G2, G4, G6, G8, G10
Griffith	−34.29	146.04	Inland	Stone fruits	Jan (Summer)	2017	G3/G13	G2, G4, G6, G8, G11
Canberra	−35.27	149.11	Inland	Stone fruits, Oranges	Jan (Summer)	2017	G3	G2
Batemans Bay	−35.70	150.19	Coastal	Stone fruits	Feb (Wet)	2017	G3/G13	G2, G4, G6, G8, G11
Bega Valley	−36.78	149.78	Coastal	Stone fruits	Feb (Wet)	2017	G3	G2
Resampled populations								
Cape Tribulation	−16.09	145.46	Coastal	Carambola	Aug (Dry)	2018	–	G2
Alice Springs	−23.69	133.89	Inland	Stone fruits	Nov (Spring)	2017	–	G2
Sydney	−33.90	151.14	Coastal	Loquats	Sept (Spring)	2017	–	G2

Climatic zones are as described in the Köppen climate classification scheme for the years 1980 to 2016 as updated by Beck et al. [85]. Populations were considered coastal if located within 50 km of the coast, and inland otherwise. The generations scored for the bioassays are presented for all populations. Additional climate data from the nearest weather station are provided in Table S1, and further details on locations are provided in Table S3

Regressing the median knockdown time against the five key weather variables yielded one significant association, a positive association with the minimum temperature of the coldest month, and this was marginally significant (*t*_10_ = 2.26, *P* = 0.047). As this variable was correlated with several other weather variables not used in the model (annual maximum temperature, annual minimum temperature, annual temperature, minimum temperature of the coldest month, and precipitation of the wettest month, Additional file 1 Fig. S1), it was unclear which aspect(s) of climate was causally involved.

The heat resistances of the Darwin, Alice Springs and Sydney populations at G2/G3 were all slightly higher than that of the long term domesticated S06 control, while the resistances of all the other populations at that point were slightly lower than that of S06 (Fig. 2). However, by G12–15 Darwin, Alice Springs and Sydney had all lost resistance and all the rescored populations then showed lower (20%) resistance than S06 (Fig. 2), although some populations showed no significant change during domestication. No significant population differences remained by G12–15 (${\chi}_8^2$ = 1.72, ϕ = 0.19, *P* > 0.05).

### Cold resistance

**Fig. 3:**
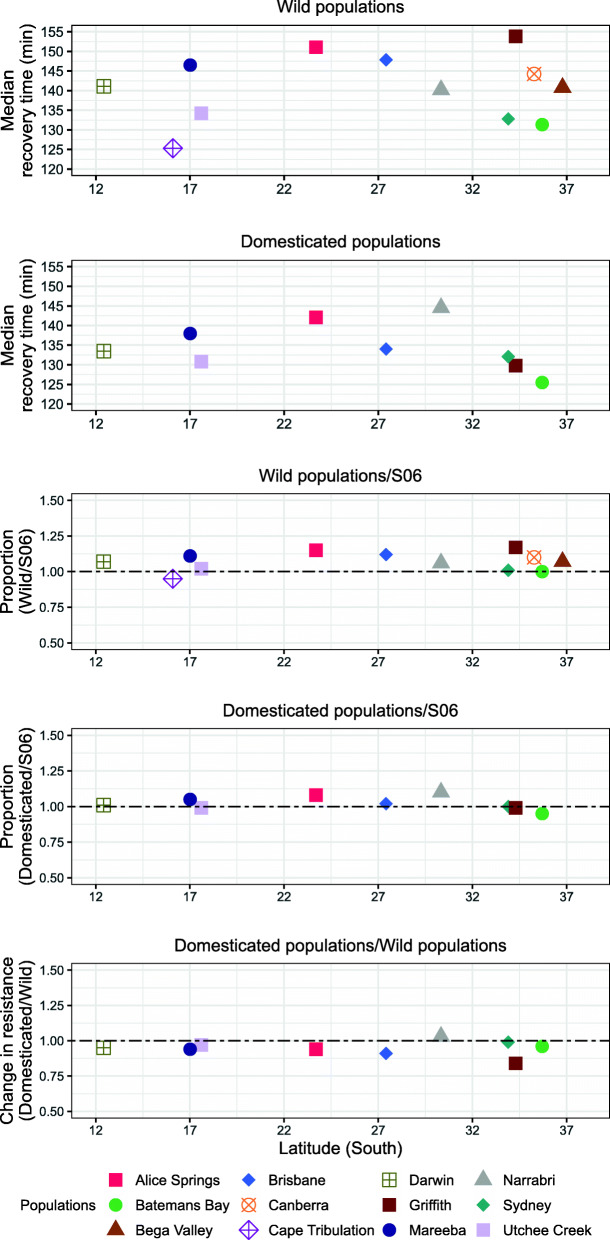
Variation in cold resistance of wild and domesticated Qfly populations. Data are presented as recovery times in minutes following exposure to 0 ± 0.5 °C for 16 h. Standard errors for recovery times were on average 18.6 and 6.1 for the wild and domesticated populations respectively. The format of the figure otherwise follows Fig. 2

Cold resistance (as measured by shorter chill coma recovery time) was not correlated with heat resistance (Table 2) and, unlike heat resistance, there were no significant differences between populations in cold resistance at G2/G3 (Fig. 3; ${\chi}_{11}^2$ = 1.55, ϕ = 0.10, *P* > 0.05). Nor were there significant population differences among the G12–15 flies (${\chi}_8^2$ = 0.87, ϕ = 0.14, *P* > 0.05), or domestication or population by domestication interaction effects (Fig. 3; ${\chi}_8^2$ = 1.12, ϕ = 0.11, *P* > 0.05, and ${\chi}_8^2$ = 0.48, ϕ = 0.11, *P* > 0.05, respectively). Neither the G2/G3 nor G12–15 data for the test populations showed consistent differences from the corresponding S06 data, although the G12–15 data were generally closer to S06 than were the G2/G3 data.

**Table 2:** Spearman correlations between stresses and wing length across wild (G2/G3) and domesticated (G10–15) samples

	Heat	Cold	Desiccation	Starvation
Heat				
Cold	0.26			
Desiccation	0.18	−0.03		
Starvation	0.30	−0.01	0.59***	
Wing length	−0.16	− 0.09	0.35*	0.05

*** P < 0.001; * *P <* 0.05

### Desiccation and starvation resistance

**Fig. 4:**
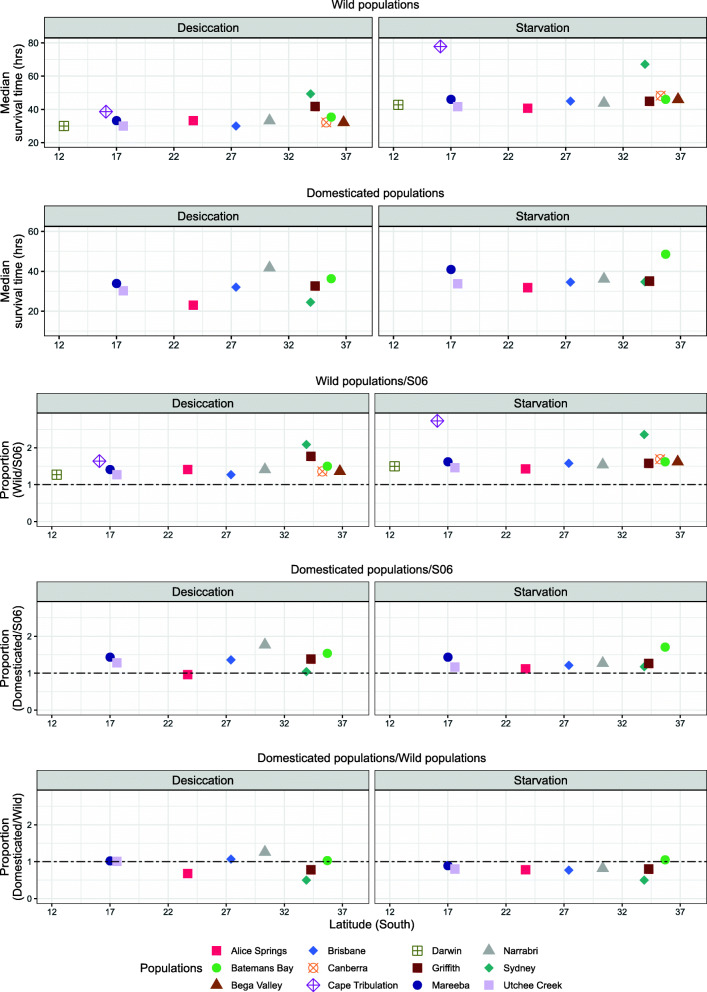
Variation in desiccation and starvation resistance of wild and domesticated Qfly populations. Data are presented as survival times in hours in the presence (desiccation) and absence (starvation) of desiccant (silica gel beads). Standard errors of survival times were on average 11.30 and 5.37 for starvation, and 5.76 and 6.08 for desiccation, in the wild and domesticated populations respectively. The format of the figure otherwise follows Fig. 2

Desiccation resistance (as measured by longer survival time under desiccating conditions) was not correlated with either heat or cold resistance (Table 2). However, similar to heat resistance, we found significant variation in desiccation resistance among the 12 G2/G3 populations (Fig. 4; ${\chi}_{11}^2$ = 6.77, ϕ = 0.08, *P* < 0.001). These differences were not explained by latitude (*t*_8_ *=* 0.97*, P* > 0.05), the coastal vs inland origins of the populations (*t*_*8*_ *=* 0.04*, P* > 0.05), the weather variables (*t*_8_ = 1.95, *P* > 0.05) or geographical distances between them (Mantel’s r = − 0.11, *P >* 0.05). The main outlier populations were Sydney, again, and to a lesser extent Griffith and Cape Tribulation, which all showed higher levels of resistance than the other populations (on average, 44.3, 21.8 and 19.4% longer survival times respectively; Fig. 4). All 12 G2/G3 test populations showed higher desiccation resistance than S06 (> 2-fold for Sydney and an average of 55% across all populations).

There was still significant variation in desiccation resistance between populations in the G10/G11 samples (${\chi}_7^2$ = 9.65, ϕ = 0.11, *P* < 0.001), and most of these samples still had substantially higher resistance than S06 (average 32% longer survival times; Fig. 4). However, the differences between populations at G10/G11 were not the same as those at G2/G3 above, which was reflected in a highly significant population by domestication interaction term (${\chi}_7^2$ = 10.33, ϕ = 0.08, *P <* 0.001). In particular, Sydney, which had the highest resistance at G2, had relatively low resistance at G10, at which point it was not significantly different from the S06 control. The three individual populations for which the G2/G3 and G10/G11 data differed significantly were Sydney and Alice Springs, both showing decreases over time (on average 54 and 68% shorter survival times respectively), and Narrabri, which showed an increase (on average 23% longer survival time; Additional file 1 Table S2).

Starvation resistance (as measured by longer survival time without access to food or water) was not correlated with heat or cold resistance but was positively correlated with desiccation resistance (Spearman’s correlation r = 0.62, *P* < 0.01; Table 2). Some correlation with the latter was expected since ‘desiccation’ was also measured in the absence of food and water, although the correlation observed implies that only 38% of the variance in the two measures was shared between them. There were significant population differences at G2/G3 in starvation resistance (${\chi}_{11}^2$ = 11.32, ϕ = 0.17, *P <* 0.05), largely due to the higher resistance of the Cape Tribulation and Sydney populations (on average 75 and 53% longer survival, respectively). However, there was no association of resistance with latitude (*t*_8_ = 0.18, *P* > 0.05), coastal vs inland origin (*t*_8_ = 0.49*, P* > 0.05), weather variables (*P >* 0.05 for all *t*_8_ tests) or geographical distance between populations (Mantel’s r = − 0.02, *P >* 0.05). The G2/G3 samples all showed higher starvation resistances than S06 (up to 2.7-fold and an average of 73% longer survival) (Fig. 4).

Starvation resistances had decreased in all except the Batemans Bay population by G10/G11 but were still higher than S06 at that point (up to 1.7-fold) (Fig. 4). However, Sydney was the only population in which the difference (decrease) was statistically significant (Additional file 1 Table S2). As with desiccation resistance, there was a significant main effect of population at G10/G11, as well as a significant population by domestication interaction (${\chi}_7^2$ = 4.91, ϕ = 0.16, *P <* 0.05 and ${\chi}_7^2$ = 4.18, ϕ = 0.16, *P <* 0.05, respectively).

### Intermediate generations

**Fig. 5:**
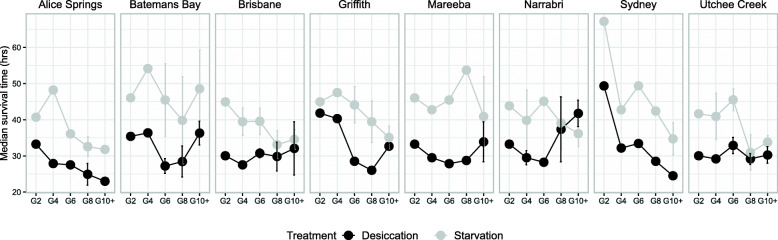
Trends over generations in the desiccation and starvation resistance in eight Qfly populations. The data are presented as normalised median survival times in hours in the presence (desiccation) and absence (starvation) of desiccant (silica gel beads). The G2 and G10+ data for each population are taken from Fig. 4

An analysis including data for the intermediate generations (*i.e.*, G4, G6 and G8) for eight populations again showed a positive correlation between desiccation and starvation resistances, albeit only explaining about 10% of variance (Spearman correlation r = 0.31, *P* < 0.01). Much of the correlation reflected a sharp drop over time in both measures in Sydney; changes in the other populations were less pronounced and not all were decreases (Fig. 5). Both desiccation and starvation resistances decreased over time in Alice Springs and Griffith, Brisbane and Utchee Creek showed drops in starvation but not desiccation resistance, Narrabri rose in desiccation but not starvation resistance, and Batemans Bay and Mareeba showed no consistent directional change in either desiccation or starvation resistance.

### Resampled populations

**Fig. 6:**
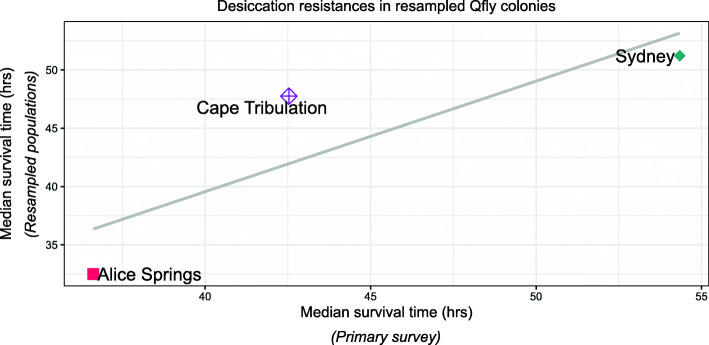
Correlation between the G2 desiccation resistances of the primary and resampled collections from Cape Tribulation, Alice Springs and Sydney. The standard errors were on average 9.02 and 6.17 for the primary and resampled collections respectively

The Sydney, Cape Tribulation and Alice Springs sites were recollected in a subsequent growing season and rescored for desiccation resistance. The initial G2 assays of these populations scored them as the highest, medium-high and medium-low respectively for desiccation resistance. The G2 assays for the recollected samples yielded resistance values that were highly correlated with those of the original collections (Fig. 6).

### Relationships to wing length

**Fig. 7:**
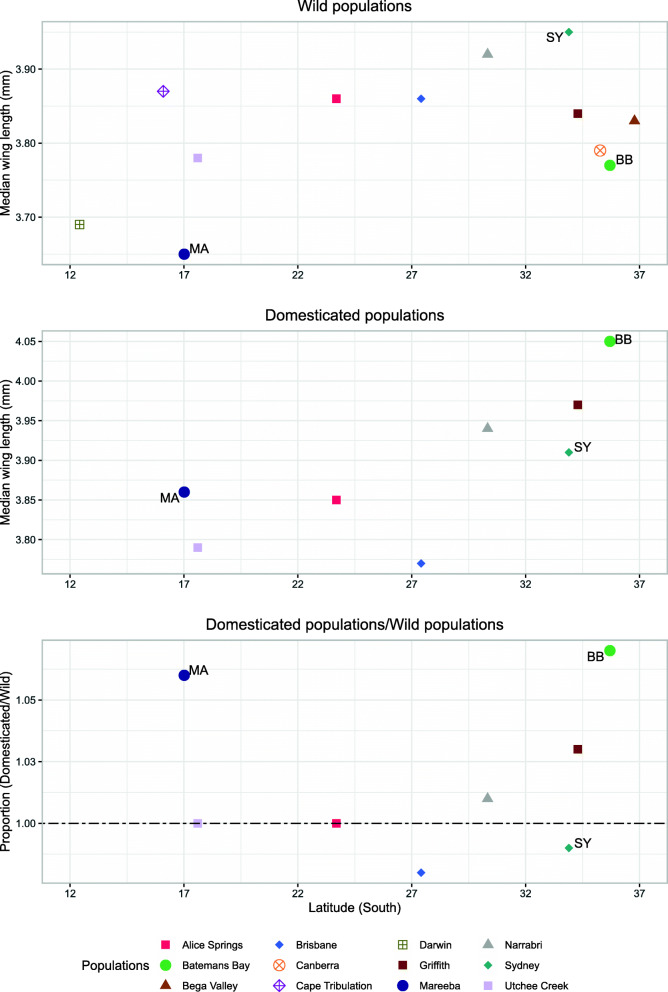
Variation in body size in wild and domesticated Qfly populations. Data are presented as median wing length in millimetres for each population in the generations designated as wild (G2) and domesticated (G10/G11). The format of the figure otherwise follows Fig. 2. BB: Batemans Bay; MA: Mareeba; SY: Sydney. Standard errors were on average 0.087 and 0.094, for the wild and domesticated population groups respectively

There were significant differences in wing length among G2/G3 populations (F_11, 329_ = 12.61, *P <* 0.001), mostly due to the longer wings of the Sydney and Narrabri samples (Fig. 7). These differences were not associated with latitude (*t*_*10*_ = 1.68, *P >* 0.05), coastal vs inland origins (*t*_*10*_ = 0.42, *P >* 0.05), or geographic distances (Mantel’s r = − 0.15, *P >* 0.05), but there was a significant association (*t*_*10*_ *=* 3.31, *P <* 0.05) with a weather variable, maximum temperature of the warmest month. As with the association of heat resistance with minimum temperature of the coldest month above, maximum temperature of the warmest month was also correlated with other weather variables not tested in this analysis (Additional file 1 Fig. S1) and we cannot determine from our results what aspect of climate was causally involved in the association.

We also found population differences in wing length in the G10/G11 samples (Fig. 7; F_8, 259_ = 41.09, *P <* 0.001), but they were generally not the same differences as at G2/G3 (F_8, 588_ = 16.51, *P <* 0.001) and there was no consistent direction to the changes seen: wing length increased in Mareeba, Griffith and Batemans Bay but decreased in Brisbane and Sydney.

**Fig. 8:**
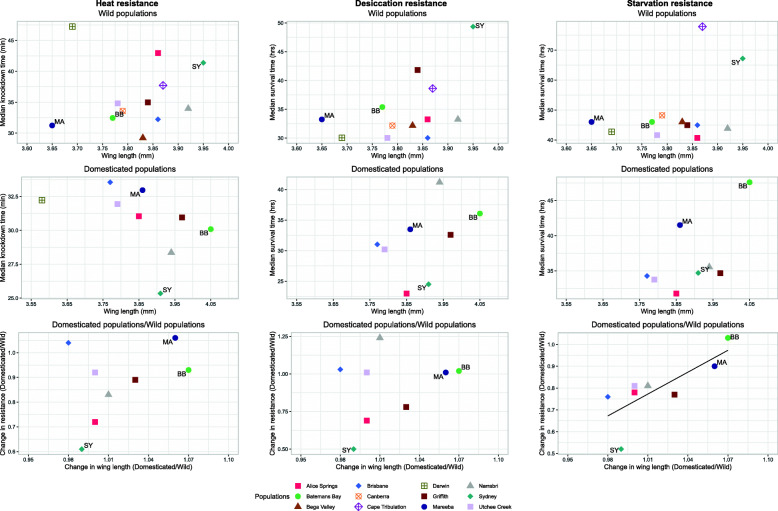
Associations between changes in stress resistances and wing length during domestication. Ratios of heat, desiccation and starvation resistances in G10–15 vs G2/G3 for each population are plotted against the corresponding ratios for wing length. The resistance data are taken from Figs. 2 and 4. BB: Batemans Bay; MA: Mareeba; SY: Sydney

Overall, neither heat, cold or desiccation resistance measurements were significantly correlated with wing length in either the G2/G3 or G10/G11 samples (all regression models with *P >* 0.05; Fig. 8). However, the change in starvation resistance in the period between the two sampling times did show a positive correlation with the change in wing length in this period (*t*_6_ = 2.91, *P* < 0.05), mostly due to the relatively large increases in both measures in Mareeba and Batemans Bay and relatively large decreases in Sydney (Fig. 8). While the corresponding correlation between the changes in desiccation resistance and wing length was not significant across all populations, it is notable that the Sydney population again changed from the highest score for both measures at G2/G3 to much lower scores for both at G10/G11 (Fig. 8).

## Discussion

We have found genetic differences in heat, desiccation and starvation but not cold resistance among the Qfly populations which were collected in 2016 and 2017 and scored at G2/G3 in a ‘common garden’ laboratory environment. The only population survey of any of these traits reported previously for Qfly was that of Meats [32] who, consistent with our findings, found no differences in cold resistance between populations sampled from Cairns (Queensland) to Nowra (NSW). Overall, the only significant correlations among the traits we measured involved desiccation resistance, which was positively correlated with both starvation resistance and body size (measured as wing length). However, one population, Sydney, stood out on several measures, being one of three populations with the highest levels of heat resistance (together with Alice Springs and Darwin) and desiccation and starvation resistance (along with Cape Tribulation and Griffith) and also being the largest in size. Importantly, we also found that the population differences in desiccation resistance were stable, inherited features of the populations, including Sydney, which we were able to resample in the subsequent year.

Unlike the situation with heat and cold resistance and wing size in *D. melanogaster* populations in eastern Australia [10, 19], we found no strong association between any of our stress resistance traits and latitude or weather variables. We acknowledge that our sample size (12 populations) was too small to detect small changes with latitude. Nevertheless, it appears that Qfly has more complex ecotypic variation in these variables in eastern Australia than does *D. melanogaster*.

Our findings concur with the climate modelling work of Yonow and Sutherst [35], which suggested that cold is less important than heat and desiccation stress in limiting the distribution of Qfly in eastern Australia. Desiccation stress may be particularly important; it has been suggested [35] that the uptake of irrigation systems has been key to the southward expansion of the species during the twentieth century, and that without irrigation this species would still be restricted to the north-eastern coast in Queensland. However, it is unclear why Sydney should be the major outlier in terms of heat, desiccation and starvation resistance and body size. Bateman [39] did include Sydney samples in his thermal stress experiments but, as noted in the Introduction, some of his samples included *B. neohumeralis*. Yu et al. [52] and Cameron et al. [53] did not find genetic differences between Sydney and Brisbane populations but their work was based on a small number of microsatellite markers which would be unlikely to influence climate stress resistance or body size. Follow-up work is needed on these traits, and their genetics, in populations between Sydney and the nearest populations we sampled to the north (Brisbane, Narrabri) and south (Batemans Bay).

While other relevant literature on Sydney samples may be lacking, in broader terms our evidence for geographic differentiation among Qfly populations is corroborated by several other lines of evidence. The microsatellite studies cited above did find differences between Northern Territory, particularly Alice Springs, populations and east coast samples [52, 53] and there is also some evidence for geographically restricted populations which are relatively less melanised than most populations (e.g. form *melas* in the Brisbane-Rockhampton area in Queensland, which is now considered a synonym species of Qfly) [4, 54, 55]. The existence of the very closely related *Bactrocera aquilonis* in northern Western Australia [54, 56] (but see also [53]) also indicates a propensity of the lineage to differentiate geographically. Similar to the ecotypic differences observed in other polyphagous fruit fly species [57, 58], our abiotic stress resistance profiling of Qfly populations also indicates the presence of genetically stable ecotypes in Australia.

We found the resistance of most populations to heat and starvation stress declined to values closer to those of the long domesticated S06 control population (~ 12 years in the laboratory at the time of testing) during the average of ten generations of domestication. Domestication effects were less consistent for desiccation resistance; decreases were observed for three populations, Sydney, Cape Tribulation and Griffith, which initially showed relatively high resistance, but others showed little change or, in a couple of cases, increases. The changes in body size were also variable and, in that case, did not show any convergence of extreme values towards the mean during domestication. The only previous study of the effects of domestication on any of these traits in Qfly was Weldon et al. [59] who found that the desiccation resistance of a recent Sydney collection was higher than that of one that had been kept in the laboratory for 20 generations. This concurs with our findings for the Sydney population. Reductions in starvation and desiccation resistance during domestication are also well documented in *Drosophila subobscura* [60, 61] and *D. melanogaster* [62]*.*

Work on a variety of insects, including some tephritid fruit flies [63, 64], has shown that various other traits also change during domestication (Hoffmann and Ross [65] for a review). Many of these, for example reductions in pre-adult development time and sexual maturation time in adults and increased fecundity, involve an increase in fitness under laboratory conditions. Some of these are also known to occur in Qfly. For instance, Meats et al. [66] found age of mating decreased after just four generations of domestication, and higher fecundity also developed subsequently. Other traits demonstrated to change as the species adapts to the laboratory include thermal preferences [67, 68], protein consumption [66], increased abundance of volatiles released during courtship [69], locomotor activity [49], and reduced dispersal rates if released into the field [50]. Gilchrist et al. [70] also found a reduction in microsatellite variability during Qfly domestication. Given the number of characters affected, it seems likely that genetic changes during domestication would be widespread across the Qfly genome.

Few of these other studies compared the effects of domestication on different populations of the species in question as we have done. One exception is Simões et al. [61] who found significant differences between *D. subobscura* populations in the rate of the decline in starvation resistance. Various other *Drosophila* studies, using genetic markers ranging from chromosome inversions through to SNPs from genome-wide resequencing, have reported some convergent allele/haplotype frequency changes among different source populations brought into a common laboratory environment but also many cases of divergent trajectories, even among replicate populations from the same source [71–73]. We concur with Santos et al. [74] that genetic variation involved in field fitness but essentially irrelevant to the laboratory environment is likely to diverge rapidly in laboratory lines of necessarily small founding populations.

The implications of our findings for Qfly SIT programmes are twofold. Firstly, our results show that genetic changes with significant deleterious effects on the performance of flies released into the field could accumulate within the timeframe in which a strain is scaled up for mass rearing and release. It will clearly be important to minimise this timeframe, or the rate of change. Secondly our results show that source populations for candidate SIT strains can vary substantially in key traits, and in the changes in those traits during the course of domestication. Thus, it would be prudent to evaluate populations from multiple sources and to explore rearing conditions for candidate strains which minimise any deterioration in the key traits during domestication. Notable here is the finding of Gilchrist and Meats [50] that intercrossing among domesticated Qfly strains can ameliorate some aspects of field fitness losses, although the practicality of implementing such a crossing scheme in a factory setting may be challenging. Also notable is the demonstration in the Mexican fruit fly *Anastrepha ludens* that directional selection regimes can be developed that increase the level of desiccation resistance of candidate SIT strains in the laboratory whilst retaining their reproductive fitness [51, 75].

Molecular genetic analyses are now needed in order to elucidate both the genetic bases for the ecotypic variation in the resistance traits we have found and the changes in these traits that occur during domestication. Such analyses could also provide the molecular markers needed for breeding and selection programmes aimed at minimising loss of resistance during domestication. Importantly a draft genome sequence is now available for Qfly which would enable these analyses [76]. Precedent work with *D. melanogaster* suggests polygenic control will be found, albeit with a few genes of major effect, and also allowing the possibility of some epigenetic effects [77].

Also now needed is a screen for any differences in the ‘plastic’ responses of Qfly populations to the various stresses. Abundant literature on various *Drosophila* species indicates that prior exposure to milder climate stress can augment climate stress resistance of individuals and that the extent of the augmentation can vary in an inherited fashion between populations [30, 78–81]. Thus, future molecular genetic work should also consider the ‘plastic’ components of the resistance phenotypes.

## Conclusions

We have found significant natural differences in heat, desiccation and starvation resistance between Qfly populations of diverse geographical origins. The fact that the differences were detected in a ‘common garden’ laboratory environment after two - three generations in the laboratory strongly suggests a genetic basis for the differences. No association with latitude or coastal vs inland origins was detected in any of the three stress-related traits, and only a relatively weak association with a climate variable was found for heat resistance and wing length. This contrasts with the strong latitudinal and climatic associations found for climate stress resistance among *D. melanogaster* and *D. serrata* populations also collected from eastern Australia and suggests complex ecotypic variation exists across the species’ range. Our data corroborate earlier evidence for ecotypic variation among Qfly populations based on microsatellite markers [52, 53, 82]. In general terms the data support the idea that the success of this pest in invading a relatively wide range of climatic environments is associated with genetic variation affecting its adaptation to different climatic conditions. We have also found significant losses of heat and desiccation resistances with ongoing domestication of some but not all of the tested populations. The differences between the populations in this respect bear out the complexity of the ecotypic variation. Both the geographic variation and the changes with domestication also have practical implications for the SIT programmes now being mounted to control Qfly [83, 84]. In particular they point to the importance of developing a strain from a relatively stress resistance base population and that procedures to preserve resistance need to be implemented from the outset of mass-rearing [42, 59, 70].

## Methods

### Fly stocks and husbandry for the primary survey

Twelve populations of Qfly were established in the laboratory from infested fruits collected from across the Australian distribution of the species during the fruit growing seasons (largely summer) of 2016/2017. The collections sites cover a broad range of the climatic conditions (following the Köppen climate classification [85]) within the species’ range (Fig. 1, Table 1 and Additional file 1 Table S1). Infested fruits were kept in the laboratory at 24–26 °C and third instar larvae were allowed to pupate in fine-grade vermiculite. The vermiculite was kept in drainage trays to avoid drenching the pupae with juice from the decomposing fruit, but in some cases it was also necessary to lightly moisten the vermiculite to reduce the risk of pupae dehydrating [86]. Pupae were sieved out of the vermiculite approximately every 10 days for three to 4 weeks after fruit collection. Adult flies were kept in Bugdorm rearing cages (31.5 × 31.5 × 31.5 cm BugDorm 43,030 Insect Rearing Cages, MegaView Science, Taiwan). Adult flies (less than 3 days old) identified as *B. tryoni* by taxonomic features detailed in Drew [54] were used to establish 1–4 replicate colonies for each population, using 80–172 flies of each sex per replicate cage (Table 1).

The colonies were then maintained for up to 15 generations under the following regimen. Adult flies were provided hydrolysed yeast (Yeast Hydrolysate Enzymatic, Cat No 103304, MP Biomedicals LLC, Australia), granulated white sugar, and water (separately ad libitum). Cages were maintained at 24-26 °C and 65 ± 5% relative humidity with 12 h light per day plus 1 h of simulated dusk and dawn. The simulated dusk (low light intensity, < 100 lux) conditions were used to induce mating [87]. Eggs were collected over 3 days from 19 to 24 days old females (6–10 h oviposition per day) using as an egg laying device, a translucent 250 mL plastic bottle (Décor, Sauce dispenser, Item No 128440) with ~ 80 small punctures on one side and a slice of apple (as an attractant) hanging inside (Additional file 1 Fig. S2). The bottle was rinsed with water to collect the eggs at the bottom (if few eggs were produced the water was strained through a nappy liner (Woolworths Homebrand) placed over the bottle mouth to collect as many eggs as possible). Up to ~ 300 eggs were transferred to 35 mL of gel diet [88] in a 90 mL round plastic container (Ø76 x 25H) (Cat. No. 01C2, Chanrol Pty Ltd.) for larval development, and the lid partially closed to prevent dehydration of the diet. The plastic container with the eggs was then placed on vermiculite in another 750–1000 mL rectangular plastic container. The lid of the container holding the diet and eggs was removed 5 days later to allow larvae to exit the diet to pupate in the vermiculite. During this time the external plastic container was covered with a nappy liner (Woolworths Homebrand) secured by a lid with an 8 cm × 14 cm hole cut out of the middle.

All populations were scored for heat, cold, desiccation and starvation resistance and wing length at generations 2 or 3 (G2 or G3) (considering the flies that emerged from the infested fruit as G0), with nine of those populations also scored in subsequent generations (Table 1). Eight of those nine populations were rescored for desiccation and starvation resistances in trials conducted every 2–3 generations until G10 or G11. The same eight domesticated populations were also rescored for heat and cold resistances and wing length once at G12–15. The ninth population, from Darwin, was rescored for heat and cold resistances only, and only at G15.

We used a domesticated line, S06, originally collected in Sydney in 2006 [70] and maintained under the same conditions as the colonies above as a control for batch effects in every resistance bioassay (see below). The phenotypic characteristics of this long-term domesticated line had stabilised well before our experiments and showed no systematic change during the course of our work.

The resistance data presented herein were based on bioassays of adult males because previous studies and our own preliminary work suggested the stress resistance phenotypes could be confounded by the reproductive history of the females, probably due to changes in their resource utilisation following mating [89, 90]. The males used were 19–24 days old when tested, at which age they would still be robust and at the peak of their fertility under laboratory conditions [91–94]. We used this age range to ensure all flies would be fully mature when tested, because wild-caught Qflies generally mature later than domesticated strains (noting that the domesticated SIT Qflies are mostly mature at 8 days) [66]. The early generation colonies were not highly productive, necessitating pooling of flies from different replicate colonies for the bioassays. However as fly fecundity increased over time [66, 70], from G6 onwards replicate colonies could be assayed independently for desiccation and starvation resistances.

Following well established precedents in the literature for various higher dipterans, we assessed thermal and desiccation resistances with heat knockdown and chill coma recovery time assays and desiccation survival time assays respectively [17, 19, 26–29, 95–97]. Our desiccation survival time assays closely followed a published method for Qfly, including the use of parallel ‘control’ assays of starvation survival times as well [46]. Our heat knockdown time and chill coma recovery time assays were based on the methods used to investigate latitudinal clines for those traits in eastern Australian *D. melanogaster* and *Drosophila serrata* [95–97].

### Heat knockdown (heat resistance) assays

Heat resistance assays were based on the protocol of Sgrò et al. [96]. Thirty adult males from each population were placed individually in 5 mL polypropylene tubes (Ref. 60.992.523, Sarstedt, Australia), submerged in a transparent Perspex water bath preheated to 42 °C, and knockdown time recorded for each fly. Pilot experiments showed 42 °C knocked down the great majority of flies in less than 1.5 h whilst still giving a broad distribution of knockdown times within that interval (Additional file 1 Fig. S3). Knockdown time was defined as the time it took a fly to become immobilised and unresponsive to illumination by an LED flash light (Mini LED Torch, Cat No. TOR9LEDBK, Officeworks, Australia). Trials were stopped after 1.5 h, and any flies not knocked down at that point (< 1% of the total) were scored with the stoppage time. The same experimental set up was used to document changes in heat resistance in domesticated colonies after 12–15 generations of laboratory adaptation (Table 1).

### Chill coma recovery (cold resistance) assays

To measure cold resistance thirty adult males from each population were placed individually in 2 mL polypropylene tubes. The tubes were then sandwiched between two identical 70-well Perspex plates (297 × 210 × 13 mm), held together by screws at the corners (Additional file 1 Fig. S4). Each unit was then sealed inside two waterproof bags and submerged in ice water (0 ± 0.5 °C) for 16 h. In a given trial, equal numbers of males of each population were randomly distributed into each unit. At the end of the 16 h the units were removed from the ice water and placed on a white topped bench at room temperature and lighting. The tubes were quickly removed, and the flies gently moved to the centre of their respective wells. A transparent, 3 mm thick Perspex sheet (297 × 210 × 3 mm) was then placed onto the top of each unit. Recovery time was defined as the time it took the flies to stand on their legs. Flies (< 1% of the total) that had not recovered at our final scoring time (300 mins after the 16 h cold treatment) were assigned the recovery time of 300 mins, following Gilbert et al. [97] and Hoffman et al. [98]. The Batemans Bay, Bega Valley, Canberra, Griffith and Mareeba populations were measured at G3, with the other populations tested at G2. The same experimental setup was used to monitor changes in cold resistance in domesticated colonies after 12–15 generations of laboratory adaptation (Table 1).

### Desiccation and starvation resistance assays

Desiccation and starvation resistances were measured following the protocol of Weldon and Taylor [46], with minor modifications. Two cohorts of 30 males from each population or colony being tested in a given trial were transferred individually to 5 mL polypropylene tubes. For one of the cohorts, the desiccation treatment, the lid of each tube contained eight silica gel beads (Stock keeping unit (SKU): SBR-P, Silica Gel Australia) which were held in place and physically separated from the fly by cotton wool. The lids for the other cohort, the starvation treatment, had cotton wool but not silica gel beads. The tubes were then returned to standard rearing conditions and mortality scored every 8 h for the following 16 h, then every 2 h for a further 76 h, and finally every 4 h until the last fly died. Flies were scored as dead if no movement was observed after flicking the tubes. A red-filtered torch light was used to score flies during the night cycles.

### Wing length measurement

Studies on a variety of insect species have found variation in climate stress resistance within the species is often correlated with body size, using body weight, wing length or wing centroid size as a proxy for body size [17, 18, 24, 46, 99, 100]. The structure and magnitude of our experiments prohibited us measuring any of these traits on treatment flies, so we measured wing length on frozen material as a surrogate, since wing length has been shown to correlate strongly with body size and weight in Qfly [101, 102]. Wing length was measured on 10 to 12 randomly selected males (frozen at the end of each generation scored, i.e., G2/G3 and G12-G15) per replicate cage for each population. The right wing of each fly was mounted onto a microscope slide. A Leica (M205 A) stereomicroscope fitted with a Leica DFC500 digital camera (Leica Microsystems) was used to photograph individual fly wings. Wing length was measured in mm from the proximal edge of the basal radial cell to the intersection of the costal and R4 + 5 veins on the margin of the wing. All measurements were done using the Leica Application Suite (V 4.12.0, Build 86) with the live interactive measurement module.

### Resampling populations for desiccation resistance

Populations from three sites were resampled across two subsequent summers and rescored for desiccation resistance at G2. Colony establishment and husbandry were as described above, and desiccation resistance was also scored essentially as above, the only exception being some minor differences in desiccant presentation for the 2017/2018 collections, as detailed in Additional file 1 Table S3. Once again S06 was also scored in each trial to enable comparisons across trials.

### Statistical analysis

As noted, resistance values for S06 flies were used to standardise the corresponding data from the experimental flies in each batch of bioassays. The structure of the experiment necessarily meant that not all populations and generations could be assayed in a single batch so, following an approach recommended by Kleynhans et al. [21] and Mitchell et al. [103], we incorporated in every batch of assays a long-established standard reference strain (S06 in our case) whose performance showed no systematic change over the course of the experiment. In our case standardisation entailed dividing the value for each tested individual by the ratio of the median of S06 values for that batch of assays over the overall median of S06 values across all batches.

All statistical analyses were carried out and figures generated using the R software and packages [104]. Relevant helper functions and packages used in the present study are listed in the Additional file 1 and additional scripts and raw data can be found at http://github.com/Angel-Popa/Qfly_abiotic_stress_resistance. Goodness of fit to the distributions assumed in the models was checked and confirmed with standard statistical diagnostics: residuals versus fitted values, distribution of standardised residuals, homogeneity of residual variance and Cook’s distances (Additional file 1 Fig. S5-S16).

Gamma generalized linear models (Gamma-GLM) with log-link transformations of the S06-standardised G2/G3 data for the four resistance variables were used to investigate population differences in those variables. The significance of population differences was then assessed by analysis of deviance. Given that the deviance statistic follows a *χ*^2^ distribution, the deviance values obtained could be tested for significance against a *χ*^2^ distribution for the appropriate degrees of freedom [105]. Significant effects were followed up by testing for specific effects of coastal vs inland collection sites, their latitudes, five climatic variables for the nearest weather stations (http://www.bom.gov.au) and geographic distances between the sites.

The five climatic variables used in these tests were the 5-year averages between 2013 and 2018 for annual rainfall, annual solar exposure, maximum temperature of the warmest month, minimum temperature of the coldest month, and rainfall for the driest month. Following Hoffmann et al. [19] these were chosen as the least correlated of a larger set of eleven variables (Additional file 1 Fig. S1). Bidirectional elimination was used to select the best regression model for each of the geographically varying resistance measures against the five key climate variables.

Mantel tests were used to test for any effect of geographic distance between collection sites on resistance differences between the populations. Euclidean distances for the distances between populations and between the median stress responses of these populations (Additional file 1 Table S4) were calculated using the base R [104] *dist* function, and the Mantel tests were carried out using the *mantel* function in the Vegan R packages, version 2.5–5 [106].

Gamma-GLM analyses with log-link transformations of S06-standardised G2/G3 and G12–15 data were used to investigate the effect of domestication on the four resistance variables. For these analyses a dummy variable was set up in which all the G2/G3 data were coded as ‘Wild’ and all the G12–15 data as ‘Domesticated’. Significant domestication by population interaction effects exposed in this analysis were followed up with post-hoc tests via the EMMEANS procedure from the EMMEANS R package version 1.3.2 [107].

For desiccation and starvation resistance, where data for three intervening generations were available, we also compared the regression slopes of the medians for each colony across generations using the EMTREND function from the EMMEANS package, version 1.3.2 [107].

Wing length data were subjected to analysis of variance (ANOVA) to test for effects of population and domestication, with Tukey post-hoc testing using EMMEANS as above. Associations between changes in resistance variables and wing length were tested by linear regression analysis.

Spearman correlation coefficients among various data sets were calculated using the Hmisc package, version 4.2–0 [108].

## Supplementary Information

**Additional file 1: Table S1.** Climatic variables from the Qfly collection sites. **Table S2**. Individual populations for which the wild (G2/G3) and domesticated (G10–15) bioassays results differed significantly. Contrast is calculated for the estimated mean response variable for each population by looking at the differences of the domesticated over the wild populations. The estimated mean of the contrast is calculated on the log-transformed data for the response variables. **Table S3.** Methodological differences between the standard desiccation resistance and that used for the resampled 2017/2018 collection. **Table S4.** Euclidean distance between site’s geographical coordinates. **Fig. S1. Correlation among 11 climatic variables.** Correlation values are presented together with asterisks indicating significance values for each correlation. ‘*’ *P* < 0.05; ‘**’ *P* < 0.01; ‘**’ *P* < 0.001. **mean.max** = Annual maximum temperature; **mean.min** = Annual minimum temperature; **mean.rain** = Annual rainfall; **mean.solar** = Annual solar exposure; **annual.temp** = Annual temperature; **max.high.temp** = Maximum temperature of the warmest month; **min.high.temp** = Minimum temperature of the warmest month; **min.low.temp** = Minimum temperature of the coldest month; **max.low.temp** = Maximum temperature of the coldest month; **ppt.dry.month** = Precipitation of the driest month; **ppt.wet.month** = Precipitation of the wettest month. **Fig. S2.** Egging device used in present study. **Fig. S3.** Results of the pilot experiment on heat knock down recovery time. **Fig. S4.** Cold resistance apparatus used in present study. **Fig. S5.** Diagnostic plots Gamma-GLM heat resistance in wild populations of the Queensland fruit fly. **Fig. S6.** Diagnostic plots Gamma-GLM heat resistance in domesticated populations of the Queensland fruit fly. **Fig. S7.** Diagnostic plots Gamma-GLM heat resistance change during domestication. **Fig. S8.** Diagnostic plots Gamma-GLM cold resistance in wild populations of the Queensland fruit fly. **Fig. S9.** Diagnostic plots Gamma-GLM cold resistance in domesticated populations of the Queensland fruit fly. **Fig. S10.** Diagnostic plots Gamma-GLM cold resistance change during domestication. **Fig. S11.** Diagnostic plots Gamma-GLM desiccation resistance in wild Qfly populations. **Fig. S12.** Diagnostic plots Gamma-GLM desiccation resistance in domesticated populations of the Queensland fruit fly. **Fig. S13.** Diagnostic plots Gamma-GLM desiccation resistance change during domestication. **Fig. S14.** Diagnostic plots Gamma-GLM starvation resistance in wild Qfly populations. **Fig. S15.** Diagnostic plots Gamma-GLM starvation resistance in domesticated populations of the Queensland fruit fly. **Fig. S16.** Diagnostic plots Gamma-GLM starvation resistance change during domestication.

## Data Availability

The data presented here are available in the Variation in stress resistance in Queensland fruit fly repository 10.6084/m9.figshare.9751634.v2

## Abbreviations

G10/G11: Generation 10 or 11
G12-G15: Generation 12 to 15
G2/G3: Generation two or three
Gamma-GLM: Gamma generalized linear models
Qfly: Queensland fruit fly
S06: Qfly collected in Sydney in 2006
SIT: Sterile Insect Technique
SNPs: Single nucleotide polymorphisms

## Supplementary Information

**Supplementary information** accompanies this paper 10.1186/s12863-020-00935-2.
