# Supplementary material for: Climate stress resistance in male Queensland fruit fly varies among populations of diverse geographic origins and changes during domestication
Source: BMC Genet. 2020 Dec 18;21(Suppl 2):135. doi: 10.1186/s12863-020-00935-2 (PMC7747409; doi:10.1186/s12863-020-00935-2)
Supplement: Supplementary file 1 — Additional file 1: Table S1. Climatic variables from the Qfly collection sites. Table S2. Individual populations for which the wild (G2/G3) and domesticated (G10–15) bioassays results differed significantly. Contrast is calculated for the estimated mean response variable for each population by looking at the differences of the domesticated over the wild populations. The estimated mean of the contrast is calculated on the log-transformed data for the response variables. Table S3. Methodological differences between the standard desiccation resistance and that used for the resampled 2017/2018 collection. Table S4. Euclidean distance between site’s geographical coordinates. Fig. S1. Correlation among 11 climatic variables. Correlation values are presented together with asterisks indicating significance values for each correlation. ‘*’ P < 0.05; ‘**’ P < 0.01; ‘**’ P < 0.001. mean.max = Annual maximum temperature; mean.min = Annual minimum temperature; mean.rain = Annual rainfall; mean.solar = Annual solar exposure; annual.temp = Annual temperature; max.high.temp = Maximum temperature of the warmest month; min.high.temp = Minimum temperature of the warmest month; min.low.temp = Minimum temperature of the coldest month; max.low.temp = Maximum temperature of the coldest month; ppt.dry.month = Precipitation of the driest month; ppt.wet.month = Precipitation of the wettest month. Fig. S2. Egging device used in present study. Fig. S3. Results of the pilot experiment on heat knock down recovery time. Fig. S4. Cold resistance apparatus used in present study. Fig. S5. Diagnostic plots Gamma-GLM heat resistance in wild populations of the Queensland fruit fly. Fig. S6. Diagnostic plots Gamma-GLM heat resistance in domesticated populations of the Queensland fruit fly. Fig. S7. Diagnostic plots Gamma-GLM heat resistance change during domestication. Fig. S8. Diagnostic plots Gamma-GLM cold resistance in wild populations of the Queensland fruit fly. Fig. S9. Diagnostic plots Gamma-GLM [file 12863_2020_935_MOESM1_ESM.zip › CRP12 ms_Additional files_suppl tables and figures.docx]

Supplementary figures and tables

# Variation in stress resistance in the Queensland fruit fly

Angel D. Popa-Baez, Siu Fai Lee, Heng Lin Yeap, Shirleen S. Prasad, Michele Schiffer, Roslyn G. Mourant, Cynthia Castro-Vargas, Owain R. Edwards, Phillip W. Taylor and John G. Oakeshott

**Contents**

### [R packages used in the statistical analyses.](#_bookmark20) 23

**List of Tables**

## [Climatic variables for the Qfly collection sites](#_bookmark0) 3

## [Methodological differences between the standard desiccation resistance assay and that used for the resampled 2017/2018 collection.](#_bookmark1) 4

## [Euclidean distance between sites’ geographical coordinates](#_bookmark2) 5

1. [**Individual populations for which the wild (G2/G3) and domesticated (G10-15) bioassay results differed significantly.** Contrast is calculated for the estimated mean response variable for each population by looking at the differences of the domesticated over the wild populations. The estimated mean of the contrast is calculated on the log-transformed data for the response variables.](#_bookmark3) 6

**List of Figures**

### [Egging device used in present study](#_bookmark4) 7

### [Results of pilot experiment on heat knock down recovery time. Data are presented as knockdown time in minutes on two different exposure temperatures for S06 flies. Significant differences of means between temperatures are reported with Wilcox test P-value.](#_bookmark5) 8

### [Cold resistance apparatus used in present study](#_bookmark6) 9

### [Diagnostic plots Gamma-GLM heat resistance in wild populations of the Queensland fruit fly.](#_bookmark7)10

### [Diagnostic plots Gamma-GLM heat resistance in domesticated populations of the Queensland fruit fly](#_bookmark8) 11

### [Diagnostic plots Gamma-GLM heat resistance change during domestication.](#_bookmark9) 12

### [Diagnostic plots Gamma-GLM cold resistance in wild populations of the Queensland fruit fly.](#_bookmark10)13

### [Diagnostic plots Gamma-GLM cold resistance in domesticated populations of the Queensland fruit fly](#_bookmark11) 14

### [Diagnostic plots Gamma-GLM cold resistance change during domestication.](#_bookmark12) 15

### [Diagnostic plots Gamma-GLM desiccation resistance in wild Qfly populations.](#_bookmark13) 16

### [Diagnostic plots Gamma-GLM desiccation resistance in domesticated populations of the Queensland fruit fly](#_bookmark14) 17

### [Diagnostic plots Gamma-GLM desiccation resistance change during domestication.](#_bookmark15) 18

### [Diagnostic plots Gamma-GLM starvation resistance in wild Qfly populations.](#_bookmark16) 19

### [Diagnostic plots Gamma-GLM starvation resistance in domesticated populations of the Queens- land fruit fly](#_bookmark17) 20

### [Diagnostic plots Gamma-GLM starvation resistance change during domestication.](#_bookmark18) 21

1. [Correlation among 11 climatic variables.](#_bookmark19) .............................................................................. 22

3

## Table S 1: Climatic variables for the Qfly collection sites

| **Population** | **mean.max** | **mean.min** | **mean.rain** | **mean.solar** | **annual.temp** | **max.high.temp** | **min.high.temp** | **min.low.temp** | **max.low.temp** | **ppt.dry.month** | **ppt.wet.month** |
| --- | --- | --- | --- | --- | --- | --- | --- | --- | --- | --- | --- |
| Darwin | 32.78 | 23.46 | 1720.52 | 21.14 | 28.12 | 38.9 | 33.3 | 24.4 | 29.7 | 0.0 | 1110.2 |
| Cape Tribulation | 29.80 | 22.32 | 1498.54 | 20.72 | 26.06 | 41.4 | 32.1 | 23.8 | 27.7 | 0.0 | 915.0 |
| Mareeba | 28.50 | 17.08 | 850.88 | 20.58 | 22.74 | 39.8 | 29.6 | 20.5 | 25.6 | 0.0 | 894.1 |
| Utchee Creek | 28.60 | 20.10 | 2947.60 | 19.14 | 24.35 | 40.4 | 31.0 | 22.5 | 29.2 | 0.0 | 2748.6 |
| Alice Springs | 29.96 | 13.42 | 237.95 | 21.16 | 21.69 | 45.2 | 37.2 | 23.1 | 32.8 | 0.0 | 356.8 |
| Brisbane | 26.92 | 16.80 | 1115.73 | 18.10 | 21.86 | 41.7 | 29.4 | 20.5 | 28.0 | 0.2 | 479.8 |
| Narrabri | 27.20 | 12.34 | 585.64 | 19.20 | 19.77 | 47.8 | 34.3 | 18.7 | 30.9 | 0.0 | 247.4 |
| Sydney | 23.86 | 15.08 | 1094.40 | 16.22 | 19.47 | 46.4 | 23.7 | 18.6 | 27.5 | 0.0 | 596.9 |
| Griffith | 24.84 | 10.68 | 417.72 | 18.10 | 17.76 | 46.0 | 32.0 | 15.2 | 30.0 | 0.0 | 257.1 |
| Canberra | 21.44 | 6.70 | 584.44 | 16.74 | 14.07 | 41.6 | 24.5 | 10.2 | 24.4 | 2.4 | 198.4 |
| Batemans Bay | 22.32 | 10.18 | 964.40 | 15.20 | 16.25 | 45.6 | 23.5 | 14.4 | 23.1 | 1.8 | 458.4 |
| Bega Valley | 21.32 | 10.32 | 826.92 | 15.04 | 15.82 | 44.2 | 23.0 | 14.0 | 22.7 | 0.0 | 358.0 |

*Note:* Variables names are indicative of the following weather variables: *mean.max*= Annual maximum temperature; *mean.min* = Annual minimum temperature; *mean.rain* = Annual rainfall; *mean.solar* = Annual solar exposure; *annual.temp* = Annual temperature; *max.high.temp* = Maximum temperature of the warmest month; *min.high.temp* = Minimum temperature of the warmest month; *min.low.temp* = Minimum temperature of the coldest month; *max.low.temp* = Maximum temperature of the coldest month; *ppt.dry.month* = Precipitation of the driest month; *ppt.wet.month* = Precipitation of the wettest month.

## Table S 2: Methodological differences between the standard desiccation resistance assay and that used for the resampled 2017/2018 collection.

### Difference in protocol First collection Resampled collection

Egg collection Egging device Baby (vine) capsicum

Larvae rearing Gel diet (Moadeli et al., 2017) Gel diet and baby (vine) capsicum Tubes 5 mL 10 mL

Desiccant 8 silica gel beads 0.5 g silica gel packet

Scoring after 16 hours Every 2 hours Every 3 hours

5

**Table S** 3: **Euclidean distance between sites’ geographical coordinates**

|  | **Alice Springs** | **Darwin** | **Sydney** | **Batemans Bay** | **Bega Valley** | **Canberra** | **Griffith** | **Mareeba** | **Brisbane** | **Cape Tribulation** | **Narrabri** | **Utchee Creek** |
| --- | --- | --- | --- | --- | --- | --- | --- | --- | --- | --- | --- | --- |
| Alice Springs | 0.00 | 11.67 | 20.05 | 20.25 | 20.59 | 19.12 | 16.12 | 13.33 | 19.37 | 13.84 | 17.22 | 13.55 |
| Darwin | 11.67 | 0.00 | 29.55 | 30.27 | 30.85 | 29.25 | 26.63 | 15.29 | 26.66 | 15.06 | 26.06 | 16.00 |
| Sydney | 20.05 | 29.55 | 0.00 | 2.04 | 3.18 | 2.45 | 5.11 | 17.83 | 6.72 | 18.69 | 3.82 | 17.09 |
| Batemans Bay | 20.25 | 30.27 | 2.04 | 0.00 | 1.16 | 1.16 | 4.38 | 19.29 | 8.72 | 20.17 | 5.39 | 18.58 |
| Bega Valley | 20.59 | 30.85 | 3.18 | 1.16 | 0.00 | 1.65 | 4.49 | 20.24 | 9.88 | 21.14 | 6.45 | 19.55 |
| Canberra | 19.12 | 29.25 | 2.45 | 1.16 | 1.65 | 0.00 | 3.22 | 18.63 | 8.73 | 19.52 | 4.99 | 17.94 |
| Griffith | 16.12 | 26.63 | 5.11 | 4.38 | 4.49 | 3.22 | 0.00 | 17.29 | 9.72 | 18.21 | 5.45 | 16.69 |
| Mareeba | 13.33 | 15.29 | 17.83 | 19.29 | 20.24 | 18.63 | 17.29 | 0.00 | 12.80 | 0.92 | 14.01 | 0.81 |
| Brisbane | 19.37 | 26.66 | 6.72 | 8.72 | 9.88 | 8.73 | 9.72 | 12.80 | 0.00 | 13.55 | 4.27 | 12.00 |
| Cape Tribulation | 13.84 | 15.06 | 18.69 | 20.17 | 21.14 | 19.52 | 18.21 | 0.92 | 13.55 | 0.00 | 14.88 | 1.60 |
| Narrabri | 17.22 | 26.06 | 3.82 | 5.39 | 6.45 | 4.99 | 5.45 | 14.01 | 4.27 | 14.88 | 0.00 | 13.28 |
| Utchee Creek | 13.55 | 16.00 | 17.09 | 18.58 | 19.55 | 17.94 | 16.69 | 0.81 | 12.00 | 1.60 | 13.28 | 0.00 |

**Table S** 4: **Individual populations for which the wild (G2/G3) and domesticated (G10-15) bioassay results differed significantly.** Contrast is calculated for the estimated mean response variable for each population by looking at the differences of the domesticated over the wild populations. The estimated mean of the contrast is calculated on the log-transformed data for the response variables.

### Population ratio SE z.ratio p.value

| **Heat** |  | | | |
| --- | --- | --- | --- | --- |
| Alice Springs | 1.09 | 0.09 | 1.11 | 0.27 |
| Batemans Bay | 1.17 | 0.09 | 2.07 | 0.04 |
| Bega Valley | NA | NA | NA | NA |
| Brisbane | 1.17 | 0.09 | 2.03 | 0.04 |
| Canberra | NA | NA | NA | NA |
| Cape Tribulation | NA | NA | NA | NA |
| Darwin | 1.10 | 0.08 | 1.23 | 0.22 |
| Griffith | 1.21 | 0.09 | 2.49 | 0.01 |
| Mareeba | 1.20 | 0.10 | 2.28 | 0.02 |
| **Desiccation**  Narrabri | 1.10 | 0.09 | 1.23 | 0.22 |
| Sydney | 1.09 | 0.08 | 1.08 | 0.28 |
| Utchee Creek | 1.14 | 0.09 | 1.71 | 0.09 |
| Alice Springs | 0.68 | 0.05 | -5.74 | 0.00 |
| Batemans Bay | 1.02 | 0.07 | 0.27 | 0.79 |
| Brisbane | 1.07 | 0.07 | 1.05 | 0.29 |
| Griffith | 0.90 | 0.06 | -1.56 | 0.12 |
| Mareeba | 0.94 | 0.06 | -0.93 | 0.35 |
| **Starvation**  Narrabri | 1.23 | 0.08 | 3.04 | 0.00 |
| Sydney | 0.54 | 0.04 | -9.37 | 0.00 |
| Utchee Creek | 1.04 | 0.07 | 0.59 | 0.55 |
| Alice Springs | 0.79 | 0.07 | -2.70 | 0.01 |
| Batemans Bay | 0.92 | 0.09 | -0.86 | 0.39 |
| Brisbane | 0.94 | 0.09 | -0.71 | 0.48 |
| Griffith | 0.83 | 0.08 | -2.10 | 0.04 |
| Mareeba | 0.95 | 0.09 | -0.59 | 0.56 |
| Narrabri | 0.91 | 0.08 | -1.05 | 0.29 |
| Sydney | 0.57 | 0.05 | -6.37 | 0.00 |
| Utchee Creek | 0.89 | 0.08 | -1.31 | 0.19 |


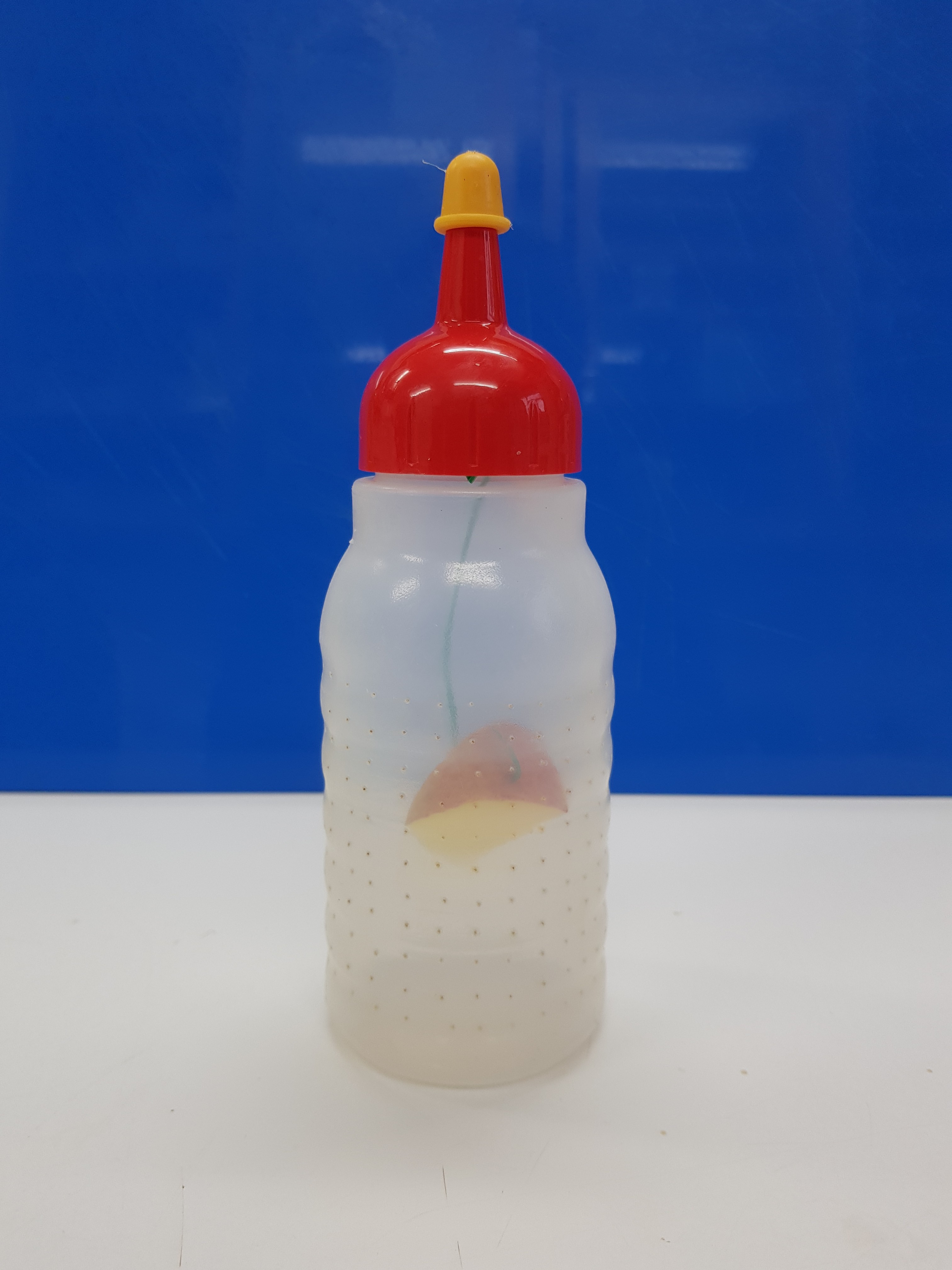


**Figure S** 1: Egging device used in present study.

80


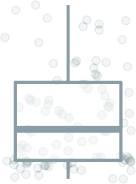

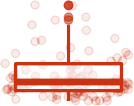


3.9e−06

60

Knockdown time (min)

Temperature

40 42 ° C


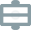

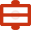


43 ° C

20

0

42 ° C 43 ° C

Temperature treatment

**Figure S** 2: Results of pilot experiment on heat knock down recovery time. Data are presented as knockdown time in minutes on two different exposure temperatures for S06 flies. Significant differences of means between temperatures are reported with Wilcox test P-value.

Well diameter = 18 mm; Gap = 10 mm; Thickness = 10 mm Thickness = 3 mm For picture screw


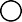

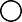

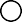

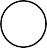

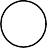

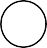

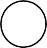

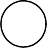

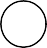

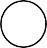

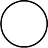

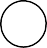

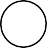

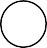

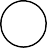

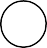

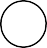

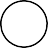

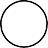

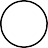

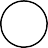

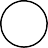

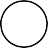

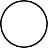

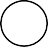

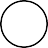

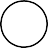

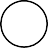

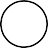

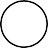

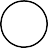

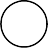

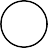

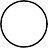

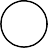

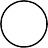

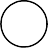

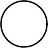

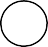

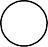

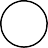

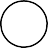

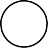

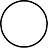

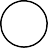

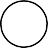

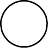

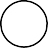

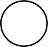

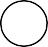

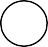

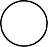

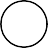

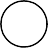

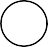

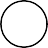

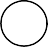

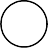

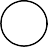

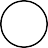

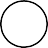

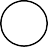

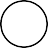

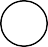

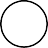

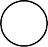

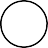

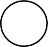

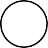

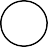

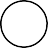

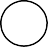

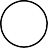

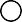

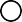

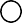

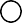


297 mm

210 mm

210 mm

297 mm

To fit one 2 mL fly vial

Glue on top to create plate with seventy 10 mm thick wells

9

**Figure S** 3: Cold resistance apparatus used in present study.

1

2

1

2

3.5 3.6 3.7 3.8 −3 −2 −1 0 1 2 3


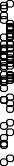

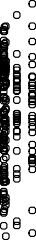

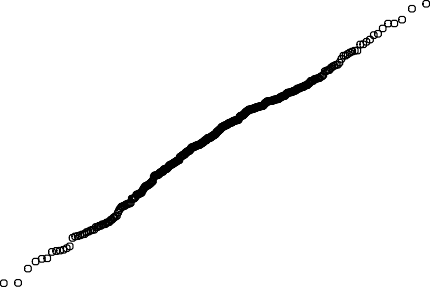


Residuals

−3

−2

−1

0

Quantiles of standard normal

−3

−2

−1

0

Linear predictor Ordered deviance residuals

Cook statistic

0.000 0.005 0.010 0.015 0.020 0.025 0.030 0.035

Cook statistic

0.000 0.005 0.010 0.015 0.020 0.025 0.030 0.035


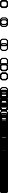

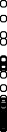

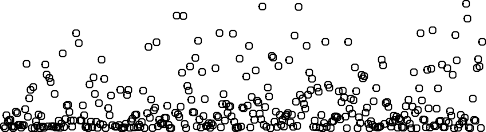


0.0346 0.0348 0.0350 0.0352 0.0354 0.0356 0 50 100 150 200 250 300 350

h/(1−h) Case

### **Figure S** 4: Diagnostic plots Gamma-GLM heat resistance in wild populations of the Queensland fruit fly.

0.00

0.01

1

2

0.00

0.01

1

2

3.40 3.45 3.50 3.55 3.60 3.65 −3 −2 −1 0 1 2 3


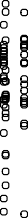

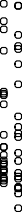

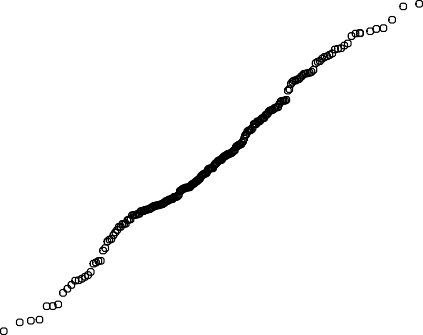


Residuals

−2

−1

0

Quantiles of standard normal

−2

−1

0

Linear predictor Ordered deviance residuals

Cook statistic

0.02

0.03

0.04

Cook statistic

0.02

0.03

0.04


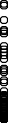

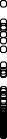

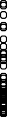

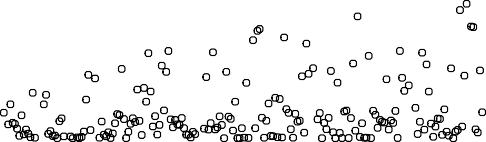


0.040 0.042 0.044 0.046 0 50 100 150 200

h/(1−h) Case

### **Figure S** 5: Diagnostic plots Gamma-GLM heat resistance in domesticated populations of the Queensland fruit fly.

0.000

0.005

2

3

0.000

0.005

2

3

3.55 3.60 3.65 3.70 3.75 3.80 3.85 −3 −2 −1 0 1 2 3


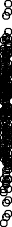

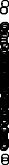

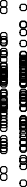

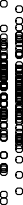

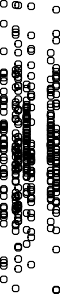


Residuals

−4

−3

−2

−1

0

1

Quantiles of standard normal

−4

−3

−2 −1

0

1

Linear predictor Ordered deviance residuals

Cook statistic

0.010

0.015

0.020

Cook statistic

0.010

0.015

0.020

0.018 0.020 0.022 0.024 0 200 400 600 800 1000

h/(1−h) Case

### **Figure S** 6: Diagnostic plots Gamma-GLM heat resistance change during domestication.

2

2

4.90 4.95 5.00 5.05 5.10 −3 −2 −1 0 1 2 3

Residuals

−4

−2

0

Quantiles of standard normal

−4

−2

0

Linear predictor Ordered deviance residuals

0.03 0.04

0.05

0.05

0.020 0.025 0.030 0.035 0.040 0 100 200 300

Cook statistic

0.00

0.01

0.02

Cook statistic

0.00

0.01

0.02 0.03 0.04

h/(1−h) Case

### **Figure S** 7: Diagnostic plots Gamma-GLM cold resistance in wild populations of the Queensland fruit fly.

2

2

4.95 5.00 5.05 5.10 −3 −2 −1 0 1 2 3

Residuals

−1

0

1

Quantiles of standard normal

−1

0

1

Linear predictor Ordered deviance residuals

0.04

0.05

0.04

0.05

0.0346 0.0348 0.0350 0.0352 0.0354 0.0356 0 50 100 150 200 250

Cook statistic

0.00

0.01

0.02

0.03

Cook statistic

0.00

0.01

0.02

0.03

h/(1−h) Case

### **Figure S** 8: Diagnostic plots Gamma-GLM cold resistance in domesticated populations of the Queensland fruit fly.

0.00

0.01

2

3

0.00

0.01

2

3

4.95 5.00 5.05 5.10 −3 −2 −1 0 1 2 3

Residuals

−2

−1

0

1

Quantiles of standard normal

−2

−1

0

1

Linear predictor Ordered deviance residuals

Cook statistic

0.02

0.03

0.04

Cook statistic

0.02

0.03

0.04

0.020 0.025 0.030 0.035 0.040 0 100 200 300 400 500

h/(1−h) Case

### **Figure S** 9: Diagnostic plots Gamma-GLM cold resistance change during domestication.

2

3

2

3

3.4 3.5 3.6 3.7 3.8 3.9 −3 −2 −1 0 1 2 3

Residuals

−3

−2

−1

0

1

Quantiles of standard normal

−3

−2

−1

0

1

Linear predictor Ordered deviance residuals

0.05

0.06

0.07

0.05

0.06

0.07

0.03448276 0.03448276 0.03448276 0.03448276 0.03448276 0 50 100 150 200 250 300 350

Cook statistic

0.00

0.01

0.02

0.03

0.04

Cook statistic

0.00

0.01

0.02

0.03 0.04

h/(1−h) Case

### **Figure S 10:** Diagnostic plots Gamma-GLM desiccation resistance in wild Qfly populations.

2

3

2

3

3.3 3.4 3.5 3.6 −3 −2 −1 0 1 2 3

Residuals

−3

−2

−1

0

1

Quantiles of standard normal

−3

−2 −1

0

1

Linear predictor Ordered deviance residuals

0.020

0.025

0.030

0.025

0.030

0.0115 0.0120 0.0125 0.0130 0 100 200 300 400 500 600 700

Cook statistic

0.000

0.005

0.010 0.015

Cook statistic

0.000

0.005

0.010 0.015 0.020

h/(1−h) Case

### **Figure S** 11: Diagnostic plots Gamma-GLM desiccation resistance in domesticated populations of the Queensland fruit fly.

2

3

2

3

3.2 3.3 3.4 3.5 3.6 3.7 3.8 −3 −2 −1 0 1 2 3

Residuals

−4

−3

−2

−1

0

1

Quantiles of standard normal

−4

−3

−2 −1

0

1

Linear predictor Ordered deviance residuals

0.03

0.04

0.03

0.04

0.01 0.02 0.03 0.04 0.05 0 200 400 600 800

Cook statistic

0.00

0.01

0.02

Cook statistic

0.00

0.01

0.02

h/(1−h) Case

### **Figure S** 12: Diagnostic plots Gamma-GLM desiccation resistance change during domestication.

3

4

3

4

3.8 3.9 4.0 4.1 4.2 −3 −2 −1 0 1 2 3

Residuals

−2

−1

0

1

2

Quantiles of standard normal

−2

−1

0

1

2

Linear predictor Ordered deviance residuals

0.10

0.12

0.10

0.12

0.0346 0.0348 0.0350 0.0352 0.0354 0.0356 0 50 100 150 200 250 300 350

Cook statistic

0.00

0.02

0.04 0.06

0.08

Cook statistic

0.00

0.02

0.04 0.06 0.08

h/(1−h) Case

### **Figure S** 13: Diagnostic plots Gamma-GLM starvation resistance in wild Qfly populations.

0.00

0.01

2

3

0.00

0.01

2

3

3.55 3.60 3.65 3.70 3.75 3.80 3.85 −3 −2 −1 0 1 2 3

Residuals

−3

−2

−1

0

1

Quantiles of standard normal

−3

−2

−1

0

1

Linear predictor Ordered deviance residuals

Cook statistic

0.02

0.03

0.04

Cook statistic

0.02

0.03

0.04

0.017 0.018 0.019 0.020 0.021 0.022 0 100 200 300 400

h/(1−h) Case

### **Figure S** 14: Diagnostic plots Gamma-GLM starvation resistance in domesticated populations of the Queensland fruit fly.

2

3

2

3

3.6 3.7 3.8 3.9 4.0 4.1 −3 −2 −1 0 1 2 3

Residuals

−3

−2

−1

0

1

Quantiles of standard normal

−3

−2

−1

0

1

Linear predictor Ordered deviance residuals

Cook statistic

0.010

0.015

0.020

Cook statistic

0.010

0.015

0.020

| 0.005 | 0.010 | 0.015 | 0.020 0.025 | 0.030 | 0.035 | 0 | 200 | 400 | 600 | 800 | 1000 | 1200 |
| --- | --- | --- | --- | --- | --- | --- | --- | --- | --- | --- | --- | --- |
|  |  |  | h/(1−h) |  |  |  |  |  | Case |  |  |  |

### **Figure S** 15: Diagnostic plots Gamma-GLM starvation resistance change during domestication.

0.000

0.005

0.000

0.005

**Figure S** 16: **Correlation among 11 climatic variables**. Correlation values are presented together with asterisks indicating significance values for each correlation. ‘*’ *P* < 0.05; ‘**’ *P* < 0.01; ‘**’ *P* < 0.001. **mean.max**= Annual maximum temperature; **mean.min** = Annual minimum temperature; **mean.rain** = Annual rainfall; **mean.solar**= Annual solar exposure; **annual.temp** = Annual temperature; **max.high.temp** = Maximum temperature of the warmest month; **min.high.temp** = Minimum temperature of the warmest month; **min.low.temp** = Minimum temperature of the coldest month; **max.low.temp** = Minimum temperature of the coldest month; **ppt.dry.month**= Precipitation of the driest month; **ppt.wet.month** = Precipitation of the wettest month.

## R packages used in the statistical analyses. R version 3.6.1 (2019-07-05)

**Platform:** x86_64-w64-mingw32/x64 (64-bit)

**attached base packages:** *grid*, *stats*, *graphics*, *grDevices*, *utils*, *datasets*, *methods* and *base*

**other attached packages:** *raster(v.2.9-5)*, *rgdal(v.1.4-4)*, *ggrepel(v.0.8.1)*, *pander(v.0.6.3)*, *Perfor- manceAnalytics(v.1.5.3)*, *xts(v.0.11-2)*, *zoo(v.1.8-6)*, *vegan(v.2.5-5)*, *permute(v.0.9-5)*, *emmeans(v.1.3.5)*,

#### boot(v.1.3-22), nortest(v.1.0-4), jtrans(v.0.2.1), Hmisc(v.4.2-0), Formula(v.1.2-3), survival(v.2.44-1.1),

*lattice(v.0.20-38)*, *xtable(v.1.8-4)*, *psych(v.1.8.12)*, *egg(v.0.4.2)*, *gridExtra(v.2.3)*, *sp(v.1.3-1)*, *cowplot(v.1.0.0)*,

*ggpubr(v.0.2)*, *magrittr(v.1.5)*, *ggridges(v.0.5.1)*, *forcats(v.0.4.0)*, *stringr(v.1.4.0)*, *dplyr(v.0.8.3)*, *purrr(v.0.3.3)*,

*readr(v.1.3.1)*, *tidyr(v.1.0.0)*, *tibble(v.2.1.3)*, *ggplot2(v.3.2.1)*, *tidyverse(v.1.2.1)* and *wesanderson(v.0.3.6)*

**loaded via a namespace (and not attached):** *TH.data(v.1.0-10)*, *colorspace(v.1.4-1)*, *ggsignif(v.0.5.0)*, *estimability(v.1.3)*, *htmlTable(v.1.13.1)*, *base64enc(v.0.1-3)*, *rstudioapi(v.0.10)*, *mvtnorm(v.1.0-10)*, *lu- bridate(v.1.7.4)*, *xml2(v.1.2.0)*, *codetools(v.0.2-16)*, *splines(v.3.6.1)*, *mnormt(v.1.5-5)*, *knitr(v.1.23)*,

#### zeallot(v.0.1.0), jsonlite(v.1.6), broom(v.0.5.2), cluster(v.2.1.0), compiler(v.3.6.1), httr(v.1.4.0), back-

*ports(v.1.1.5)*, *assertthat(v.0.2.1)*, *Matrix(v.1.2-17)*, *lazyeval(v.0.2.2)*, *cli(v.1.1.0)*, *acepack(v.1.4.1)*, *html-*

*tools(v.0.3.6)*, *tools(v.3.6.1)*, *coda(v.0.19-2)*, *gtable(v.0.3.0)*, *glue(v.1.3.1)*, *reshape2(v.1.4.3)*, *Rcpp(v.1.0.3)*,

*cellranger(v.1.1.0)*, *vctrs(v.0.2.0)*, *nlme(v.3.1-140)*, *xfun(v.0.7)*, *rvest(v.0.3.4)*, *lifecycle(v.0.1.0)*, *MASS(v.7.3-*

*51.4)*, *scales(v.1.0.0)*, *hms(v.0.4.2)*, *parallel(v.3.6.1)*, *sandwich(v.2.5-1)*, *RColorBrewer(v.1.1-2)*, *yaml(v.2.2.0)*,

*rpart(v.4.1-15)*, *latticeExtra(v.0.6-28)*, *stringi(v.1.4.3)*, *checkmate(v.1.9.3)*, *rlang(v.0.4.2)*, *pkgcon-*

*fig(v.2.0.3)*, *evaluate(v.0.14)*, *htmlwidgets(v.1.3)*, *labeling(v.0.3)*, *tidyselect(v.0.2.5)*, *plyr(v.1.8.4)*, *R6(v.2.4.1)*,

*generics(v.0.0.2)*, *multcomp(v.1.4-10)*, *pillar(v.1.4.2)*, *haven(v.2.1.0)*, *foreign(v.0.8-72)*, *withr(v.2.1.2)*,

*mgcv(v.1.8-28)*, *nnet(v.7.3-12)*, *modelr(v.0.1.4)*, *crayon(v.1.3.4)*, *rmarkdown(v.1.16)*, *readxl(v.1.3.1)*,

*data.table(v.1.12.2)*, *digest(v.0.6.23)*, *webshot(v.0.5.1)*, *munsell(v.0.5.0)*, *viridisLite(v.0.3.0)*, *kableEx- tra(v.1.1.0)* and *quadprog(v.1.5-7)*

23
