# Supplementary material for: Climate stress resistance in male Queensland fruit fly varies among populations of diverse geographic origins and changes during domestication
Source: BMC Genet. 2020 Dec 18;21(Suppl 2):135. doi: 10.1186/s12863-020-00935-2 (PMC7747409; doi:10.1186/s12863-020-00935-2)
Supplement: Supplementary file 1 — Additional file 1: Table S1. Climatic variables from the Qfly collection sites. Table S2. Individual populations for which the wild (G2/G3) and domesticated (G10–15) bioassays results differed significantly. Contrast is calculated for the estimated mean response variable for each population by looking at the differences of the domesticated over the wild populations. The estimated mean of the contrast is calculated on the log-transformed data for the response variables. Table S3. Methodological differences between the standard desiccation resistance and that used for the resampled 2017/2018 collection. Table S4. Euclidean distance between site’s geographical coordinates. Fig. S1. Correlation among 11 climatic variables. Correlation values are presented together with asterisks indicating significance values for each correlation. ‘*’ P < 0.05; ‘**’ P < 0.01; ‘**’ P < 0.001. mean.max = Annual maximum temperature; mean.min = Annual minimum temperature; mean.rain = Annual rainfall; mean.solar = Annual solar exposure; annual.temp = Annual temperature; max.high.temp = Maximum temperature of the warmest month; min.high.temp = Minimum temperature of the warmest month; min.low.temp = Minimum temperature of the coldest month; max.low.temp = Maximum temperature of the coldest month; ppt.dry.month = Precipitation of the driest month; ppt.wet.month = Precipitation of the wettest month. Fig. S2. Egging device used in present study. Fig. S3. Results of the pilot experiment on heat knock down recovery time. Fig. S4. Cold resistance apparatus used in present study. Fig. S5. Diagnostic plots Gamma-GLM heat resistance in wild populations of the Queensland fruit fly. Fig. S6. Diagnostic plots Gamma-GLM heat resistance in domesticated populations of the Queensland fruit fly. Fig. S7. Diagnostic plots Gamma-GLM heat resistance change during domestication. Fig. S8. Diagnostic plots Gamma-GLM cold resistance in wild populations of the Queensland fruit fly. Fig. S9. Diagnostic plots Gamma-GLM [file 12863_2020_935_MOESM1_ESM.zip › CRP12 ms_Additional File 1_20200921.docx]

Additional file 1 of

Climate stress resistance in male Queensland fruit fly varies among populations of diverse geographic origins and changes during domestication

Ángel-David Popa-Báez, Siu Fai Lee, Heng Lin Yeap, Shirleen S. Prasad, Michele Schiffer, Roslyn G. Mourant, Cynthia Castro-Vargas, Owain R. Edwards, Phillip W. Taylor and John G. Oakeshott

**List of Tables**

1. [Table S1: Climatic variables for the Qfly collection sites 2](#_Toc50637782)
2. [Table S2: Individual populations for which the wild (G2/G3) and domesticated (G10-15) bioassay results differed significantly. 3](#_Toc50637783)
3. [Table S3: Methodological differences between the standard desiccation resistance assay and that used for the resampled 2017/2018 collection. 4](#_Toc50637784)
4. [Table S4: Euclidean distance between sites’ geographical coordinates 5](#_Toc50637785)

**List of Figures**

1. [Figure S1: Correlation among 11 climatic variables. 6](#_Toc50592430)
2. [Figure S2: Egging device used in present study. 7](#_Toc50592431)
3. [Figure S3: Results of the pilot experiment on heat knock down recovery time. 8](#_Toc50592432)
4. [Figure S4: Cold resistance apparatus used in present study. 9](#_Toc50592433)
5. [Figure S5: Diagnostic plots Gamma-GLM heat resistance in wild populations of the Queensland fruit fly. 10](#_Toc50592434)
6. [Figure S6: Diagnostic plots Gamma-GLM heat resistance in domesticated populations of the Queensland fruit fly. 11](#_Toc50592435)
7. [Figure S7: Diagnostic plots Gamma-GLM heat resistance change during domestication. 12](#_Toc50592436)
8. [Figure S8: Diagnostic plots Gamma-GLM cold resistance in wild populations of the Queensland fruit fly. 13](#_Toc50592437)
9. [Figure S9: Diagnostic plots Gamma-GLM cold resistance in domesticated populations of the Queensland fruit fly. 14](#_Toc50592438)
10. [Figure S10: Diagnostic plots Gamma-GLM cold resistance change during domestication. 15](#_Toc50592439)
11. [Figure S11: Diagnostic plots Gamma-GLM desiccation resistance in wild Qfly populations. 16](#_Toc50592440)
12. [Figure S12: Diagnostic plots Gamma-GLM desiccation resistance in domesticated populations of the Queensland fruit fly. 17](#_Toc50592441)
13. [Figure S13: Diagnostic plots Gamma-GLM desiccation resistance change during domestication. 18](#_Toc50592442)
14. [Figure S14: Diagnostic plots Gamma-GLM starvation resistance in wild Qfly populations. 19](#_Toc50592443)
15. [Figure S15: Diagnostic plots Gamma-GLM starvation resistance in domesticated populations of the Queensland fruit fly. 20](#_Toc50592444)
16. [Figure S16: Diagnostic plots Gamma-GLM starvation resistance change during domestication. 21](#_Toc50592445)

**Contents**

[**R packages used in the statistical analyses.** 22](#_Toc50592721)

Table S1: Climatic variables for the Qfly collection sites

| **Population** | **mean.max** | **mean.min** | **mean.rain** | **mean.solar** | **annual.temp** | **max.high.temp** | **min.high.temp** | **min.low.temp** | **max.low.temp** | **ppt.dry.month** | **ppt.wet.month** |
| --- | --- | --- | --- | --- | --- | --- | --- | --- | --- | --- | --- |
| Darwin | 32.78 | 23.46 | 1720.52 | 21.14 | 28.12 | 38.9 | 33.3 | 24.4 | 29.7 | 0.0 | 1110.2 |
| Cape Tribulation | 29.80 | 22.32 | 1498.54 | 20.72 | 26.06 | 41.4 | 32.1 | 23.8 | 27.7 | 0.0 | 915.0 |
| Mareeba | 28.50 | 17.08 | 850.88 | 20.58 | 22.74 | 39.8 | 29.6 | 20.5 | 25.6 | 0.0 | 894.1 |
| Utchee Creek | 28.60 | 20.10 | 2947.60 | 19.14 | 24.35 | 40.4 | 31.0 | 22.5 | 29.2 | 0.0 | 2748.6 |
| Alice Springs | 29.96 | 13.42 | 237.95 | 21.16 | 21.69 | 45.2 | 37.2 | 23.1 | 32.8 | 0.0 | 356.8 |
| Brisbane | 26.92 | 16.80 | 1115.73 | 18.10 | 21.86 | 41.7 | 29.4 | 20.5 | 28.0 | 0.2 | 479.8 |
| Narrabri | 27.20 | 12.34 | 585.64 | 19.20 | 19.77 | 47.8 | 34.3 | 18.7 | 30.9 | 0.0 | 247.4 |
| Sydney | 23.86 | 15.08 | 1094.40 | 16.22 | 19.47 | 46.4 | 23.7 | 18.6 | 27.5 | 0.0 | 596.9 |
| Griffith | 24.84 | 10.68 | 417.72 | 18.10 | 17.76 | 46.0 | 32.0 | 15.2 | 30.0 | 0.0 | 257.1 |
| Canberra | 21.44 | 6.70 | 584.44 | 16.74 | 14.07 | 41.6 | 24.5 | 10.2 | 24.4 | 2.4 | 198.4 |
| Batemans Bay | 22.32 | 10.18 | 964.40 | 15.20 | 16.25 | 45.6 | 23.5 | 14.4 | 23.1 | 1.8 | 458.4 |
| Bega Valley | 21.32 | 10.32 | 826.92 | 15.04 | 15.82 | 44.2 | 23.0 | 14.0 | 22.7 | 0.0 | 358.0 |
| *Note:* Variables names are indicative of the following weather variables: *mean.max*= Annual maximum temperature; *mean.min* = Annual minimum temperature; *mean.rain* = Annual rainfall; *mean.solar* = Annual solar exposure; *annual.temp* = Annual temperature; *max.high.temp* = Maximum temperature of the warmest month; *min.high.temp* = Minimum temperature of the warmest month; *min.low.temp* = Minimum temperature of the coldest month; *max.low.temp* = Maximum temperature of the coldest month; *ppt.dry.month* = Precipitation of the driest month; *ppt.wet.month* = Precipitation of the wettest month. | | | | | | | | | | | |

Table S2: Individual populations for which the wild (G2/G3) and domesticated (G10-15) bioassay results differed significantly. Contrast is calculated for the estimated mean response variable for each population by looking at the differences of the domesticated over the wild populations. The estimated mean of the contrast is calculated on the log-transformed data for the response variables.

| Population | ratio | SE | Z.ratio | p.value |
| --- | --- | --- | --- | --- |
| **Heat** |  |  |  |  |
| Alice Springs | 1.09 | 0.09 | 1.11 | 0.27 |
| Batemans Bay | 1.17 | 0.09 | 2.07 | 0.04 |
| Bega Valley | NA | NA | NA | NA |
| Brisbane | 1.17 | 0.09 | 2.03 | 0.04 |
| Canberra | NA | NA | NA | NA |
| Cape Tribulation | NA | NA | NA | NA |
| Darwin | 1.10 | 0.08 | 1.23 | 0.22 |
| Griffith | 1.21 | 0.09 | 2.49 | 0.01 |
| Mareeba | 1.20 | 0.10 | 2.28 | 0.02 |
| Narrabri | 1.10 | 0.09 | 1.23 | 0.22 |
| Sydney | 1.09 | 0.08 | 1.08 | 0.28 |
| Utchee Creek | 1.14 | 0.09 | 1.71 | 0.09 |
| **Desiccation** |  |  |  |  |
| Alice Springs | 0.68 | 0.05 | -5.74 | 0.00 |
| Batemans Bay | 1.02 | 0.07 | 0.27 | 0.79 |
| Brisbane | 1.07 | 0.07 | 1.05 | 0.29 |
| Griffith | 0.90 | 0.06 | -1.56 | 0.12 |
| Mareeba | 0.94 | 0.06 | -0.93 | 0.35 |
| Narrabri | 1.23 | 0.08 | 3.04 | 0.00 |
| Sydney | 0.54 | 0.04 | -9.37 | 0.00 |
| Utchee Creek | 1.04 | 0.07 | 0.59 | 0.55 |
| **Heat** |  |  |  |  |
| Alice Springs | 0.79 | 0.07 | -2.79 | 0.01 |
| Batemans Bay | 0.92 | 0.09 | -0.86 | 0.39 |
| Brisbane | 0.94 | 0.09 | -0.71 | 0.48 |
| Griffith | 0.83 | 0.08 | -2.10 | 0.04 |
| Mareeba | 0.95 | 0.09 | -0.59 | 0.56 |
| Narrabri | 0.91 | 0.08 | -1.05 | 0.29 |
| Sydney | 0.57 | 0.05 | -6.37 | 0.00 |
| Utchee Creek | 0.89 | 0.08 | -1.31 | 0.19 |

Table S3: Methodological differences between the standard desiccation resistance assay and that used for the resampled 2017/2018 collection.

| Difference in protocol | First collection | Resampled collection |
| --- | --- | --- |
| Egg collection | Egging device | Baby (vine) capsicum |
| Larvae rearing | Gel diet (Moadeli et al., 2017) | Gel diet and baby (vine) capsicum |
| Tubes | 5 mL | 10 mL |
| Desiccant | 8 silica gel beads | 0.5g silica gel packet |
| Scoring after 16 hours | Every 2 hours | Every 3 hours |

Table S4: Euclidean distance between sites’ geographical coordinates

|  | **Alice Springs** | **Darwin** | **Sydney** | **Batemans Bay** | **Bega Valley** | **Canberra** | **Griffith** | **Mareeba** | **Brisbane** | **Cape Tribulation** | **Narrabri** | **Utchee Creek** |
| --- | --- | --- | --- | --- | --- | --- | --- | --- | --- | --- | --- | --- |
| **Alice Springs** | 0.00 | 11.67 | 20.05 | 20.25 | 20.59 | 19.12 | 16.12 | 13.33 | 19.37 | 13.84 | 17.22 | 13.55 |
| **Darwin** | 11.67 | 0.00 | 29.55 | 30.27 | 30.85 | 29.25 | 26.63 | 15.29 | 26.66 | 15.06 | 26.06 | 16.00 |
| **Sydney** | 20.05 | 29.55 | 0.00 | 2.04 | 3.18 | 2.45 | 5.11 | 17.83 | 6.72 | 18.69 | 3.82 | 17.09 |
| **Batemans Bay** | 20.25 | 30.27 | 2.04 | 0.00 | 1.16 | 1.16 | 4.38 | 19.29 | 8.72 | 20.17 | 5.39 | 18.58 |
| **Bega Valley** | 20.59 | 30.85 | 3.18 | 1.16 | 0.00 | 1.65 | 4.49 | 20.24 | 9.88 | 21.14 | 6.45 | 19.55 |
| **Canberra** | 19.12 | 29.25 | 2.45 | 1.16 | 1.65 | 0.00 | 3.22 | 18.63 | 8.73 | 19.52 | 4.99 | 17.94 |
| **Griffith** | 16.12 | 26.63 | 5.11 | 4.38 | 4.49 | 3.22 | 0.00 | 17.29 | 9.72 | 18.21 | 5.45 | 16.69 |
| **Mareeba** | 13.33 | 15.29 | 17.83 | 19.29 | 20.24 | 18.63 | 17.29 | 0.00 | 12.80 | 0.92 | 14.01 | 0.81 |
| **Brisbane** | 19.37 | 26.66 | 6.72 | 8.72 | 9.88 | 8.73 | 9.72 | 12.80 | 0.00 | 13.55 | 4.27 | 12.00 |
| **Cape Tribulation** | 13.84 | 15.06 | 18.69 | 20.17 | 21.14 | 19.52 | 18.21 | 0.92 | 13.55 | 0.00 | 14.88 | 1.60 |
| **Narrabri** | 17.22 | 26.06 | 3.82 | 5.39 | 6.45 | 4.99 | 5.45 | 14.01 | 4.27 | 14.88 | 0.00 | 13.28 |
| **Utchee Creek** | 13.55 | 16.00 | 17.09 | 18.58 | 19.55 | 17.94 | 16.69 | 0.81 | 12.00 | 1.60 | 13.28 | 0.00 |


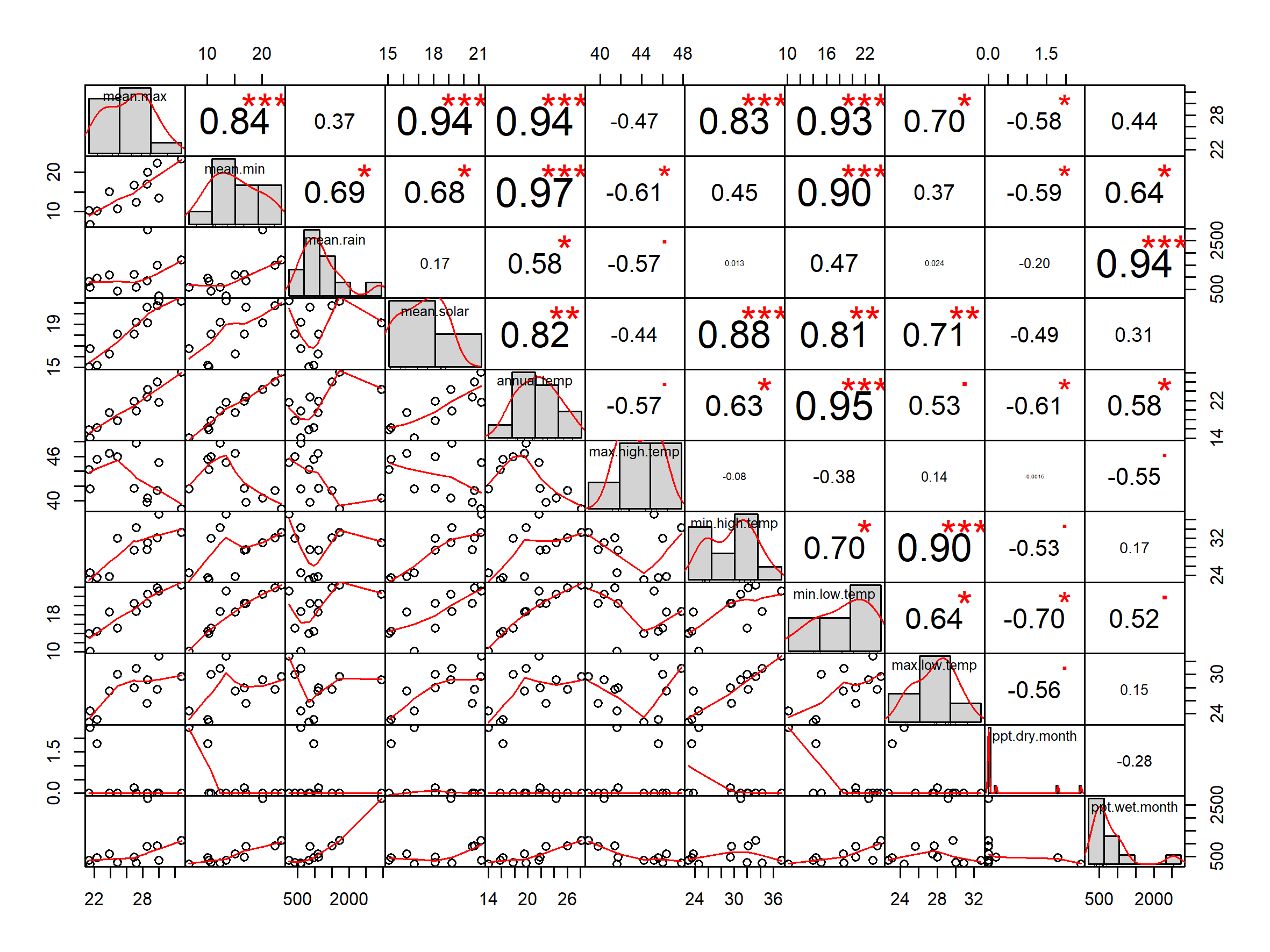


**Figure S1: Correlation among 11 climatic variables.** Correlation values are presented together with asterisks indicating significance values for each correlation. ‘*’ *P* < 0.05; ‘**’ *P* < 0.01; ‘**’ *P* < 0.001. **mean.max**= Annual maximum temperature; **mean.min** = Annual minimum temperature; **mean.rain** = Annual rainfall; **mean.solar**= Annual solar exposure; **annual.temp** = Annual temperature; **max.high.temp** = Maximum temperature of the warmest month; **min.high.temp** = Minimum temperature of the warmest month; **min.low.temp** = Minimum temperature of the coldest month; **max.low.temp** = Minimum temperature of the coldest month; **ppt.dry.month**= Precipitation of the driest month; **ppt.wet.month** = Precipitation of the wettest month.


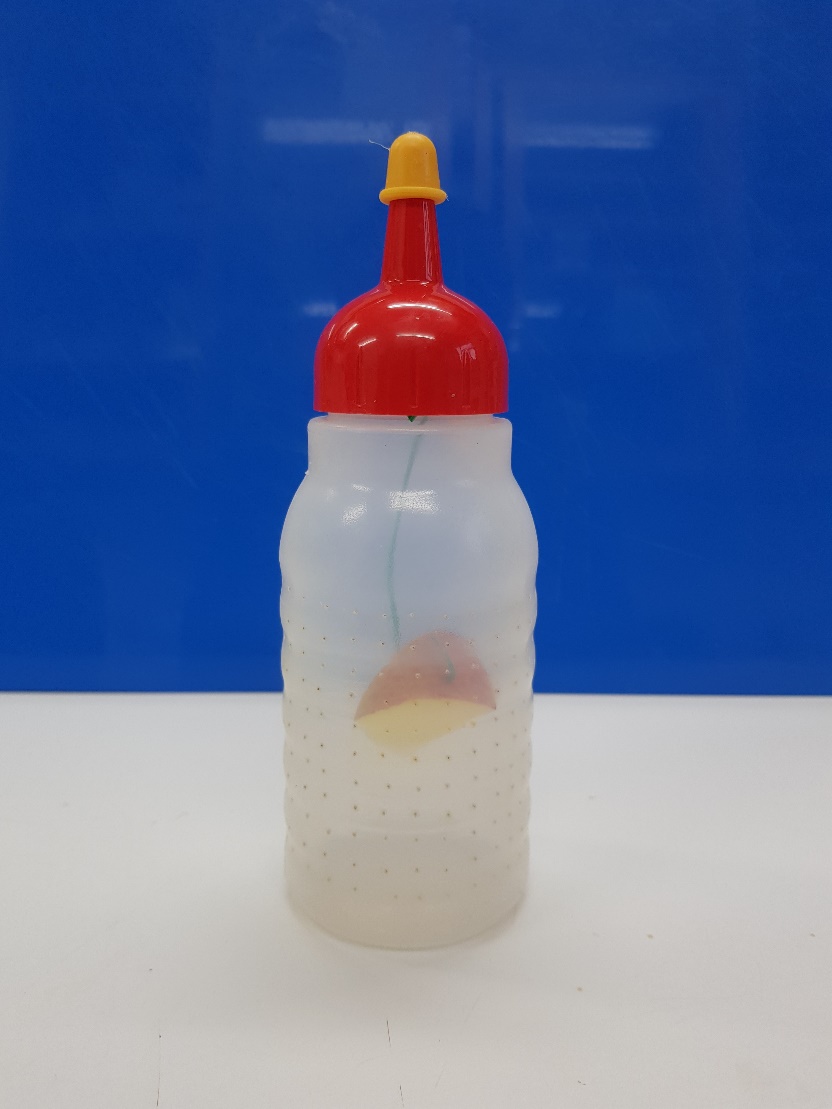


Figure S2: Egging device used in present study.


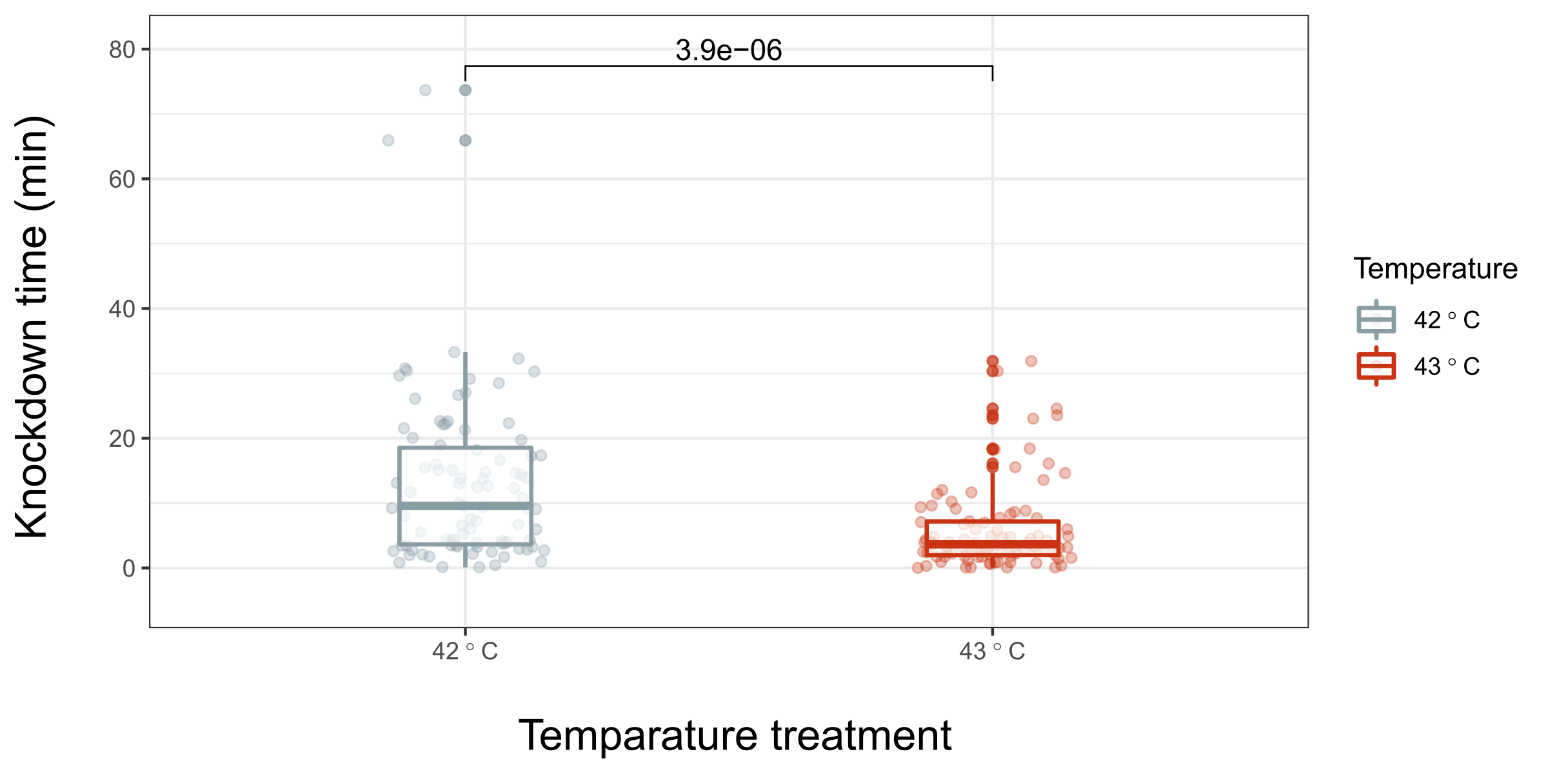


Figure S3: Results of the pilot experiment on heat knock down recovery time.


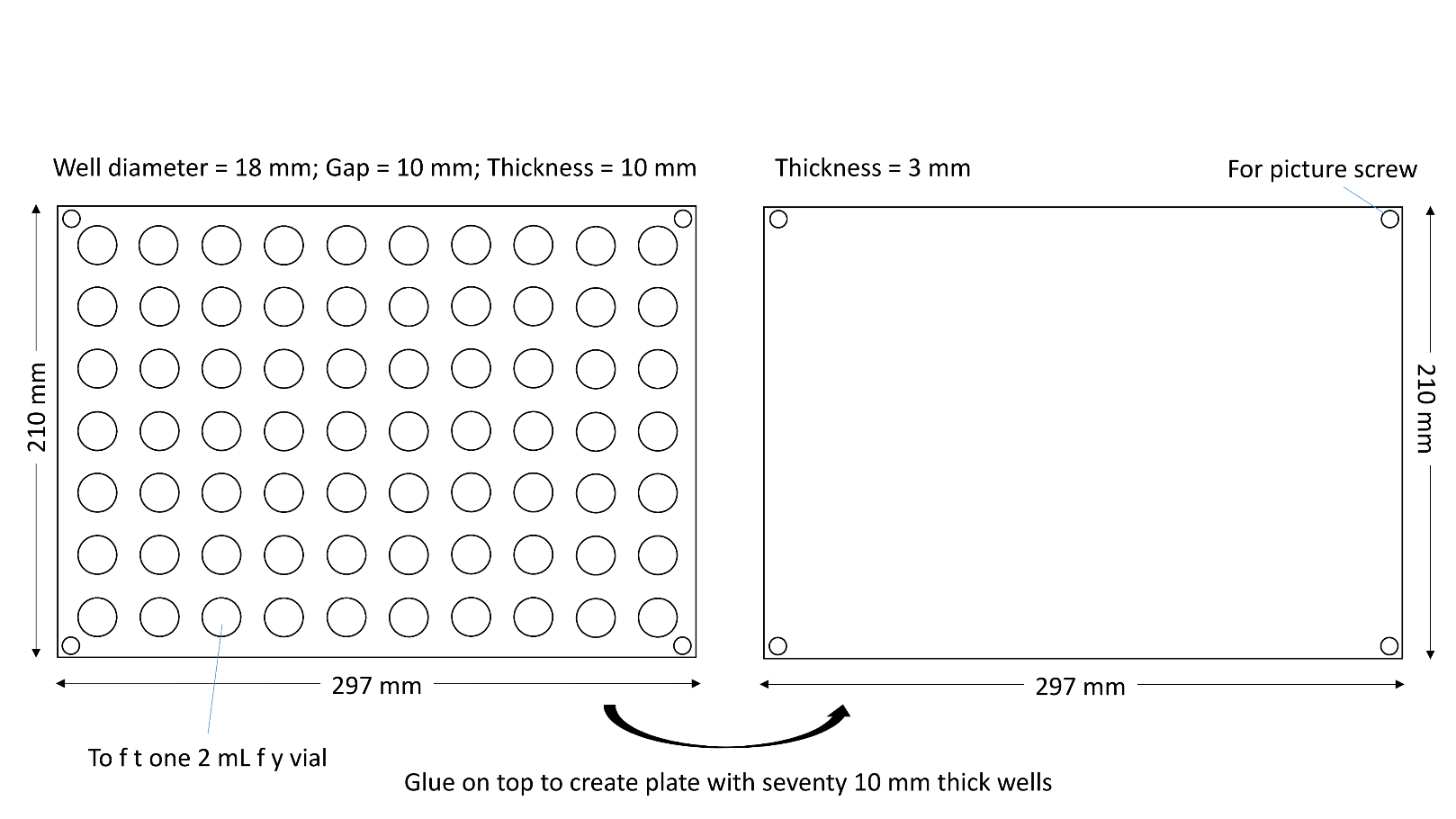


Figure S4: Cold resistance apparatus used in present study.


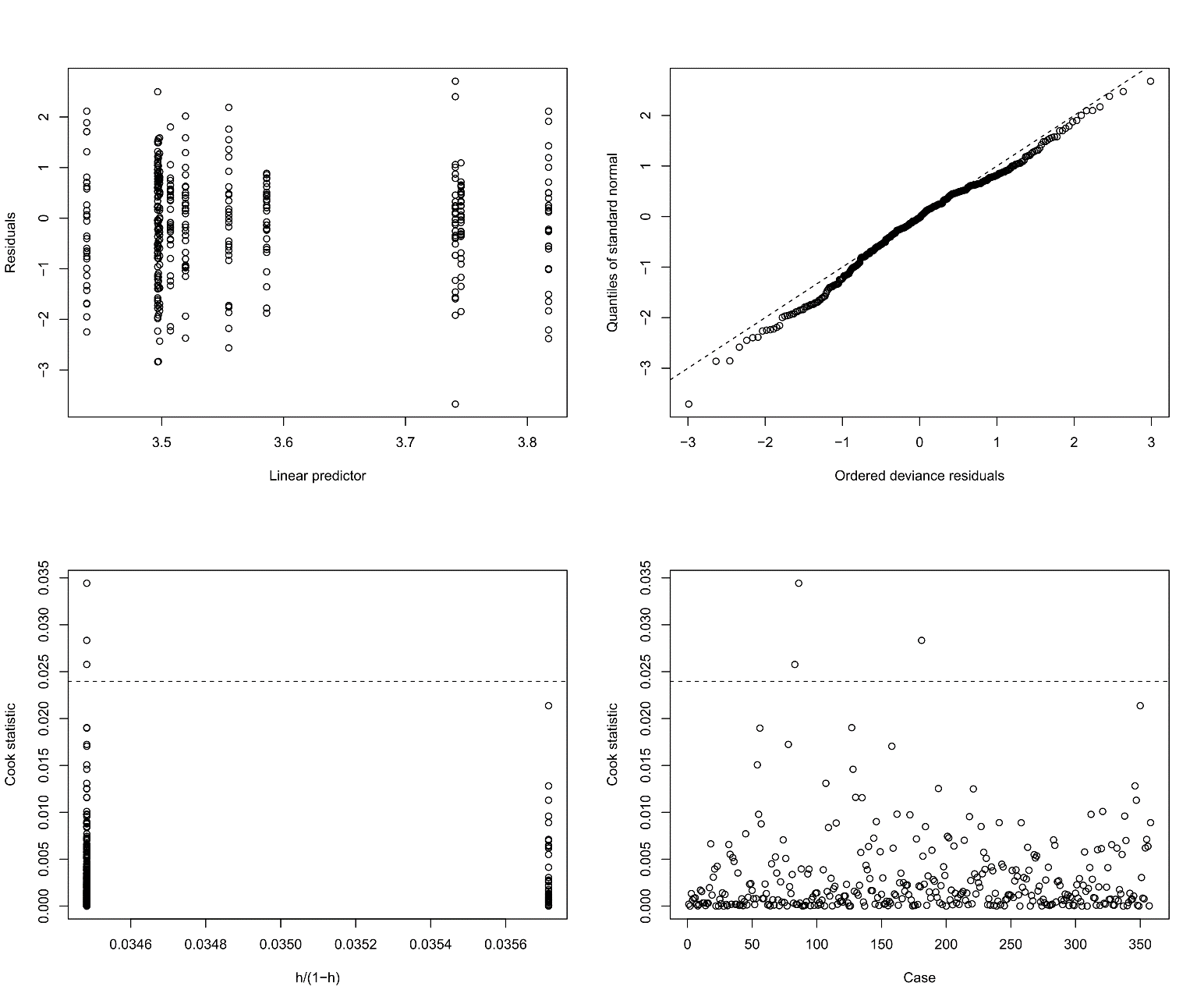


Figure S5: Diagnostic plots Gamma-GLM heat resistance in wild populations of the Queensland fruit fly.


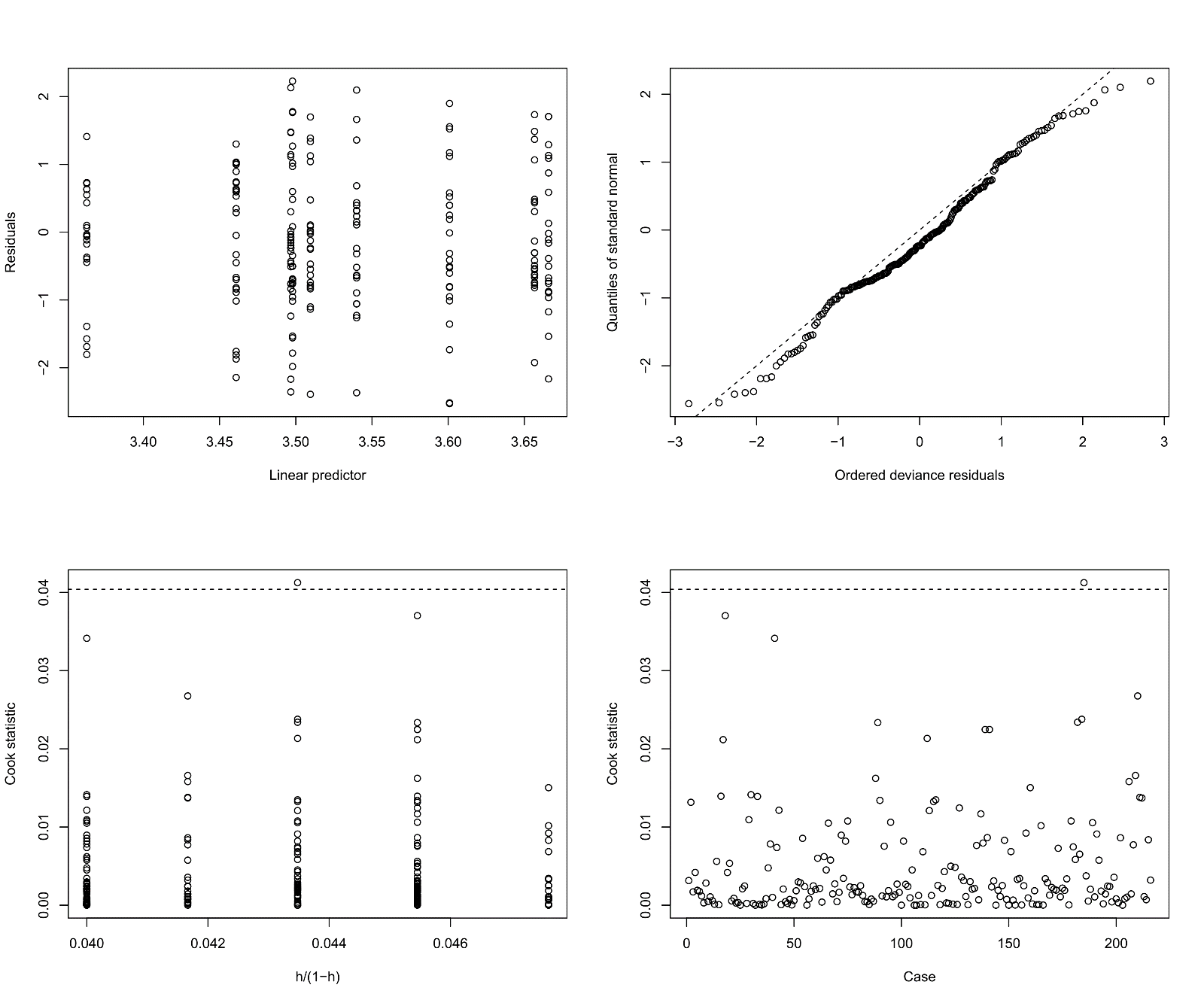


Figure S6: Diagnostic plots Gamma-GLM heat resistance in domesticated populations of the Queensland fruit fly.


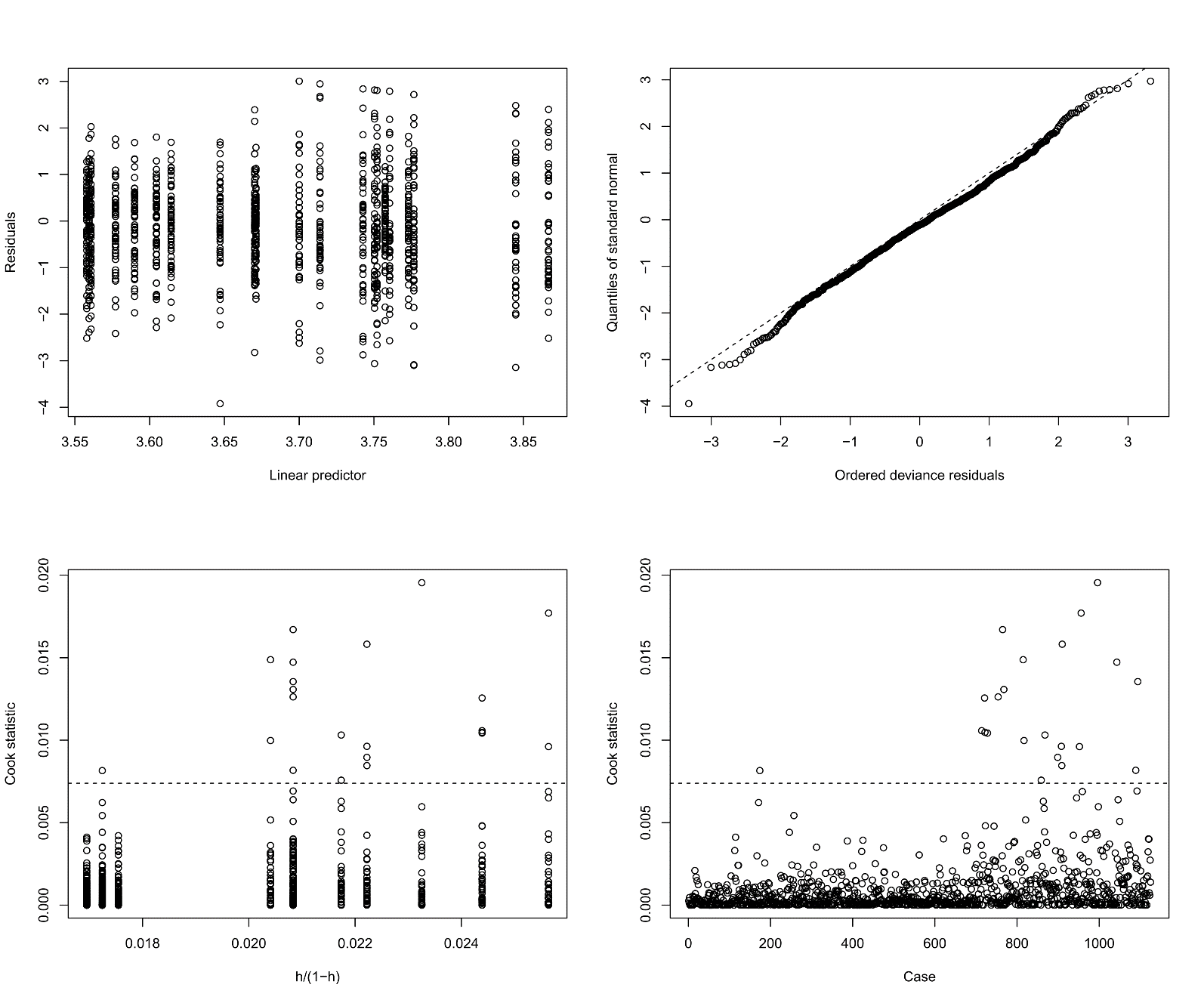


Figure S7: Diagnostic plots Gamma-GLM heat resistance change during domestication.


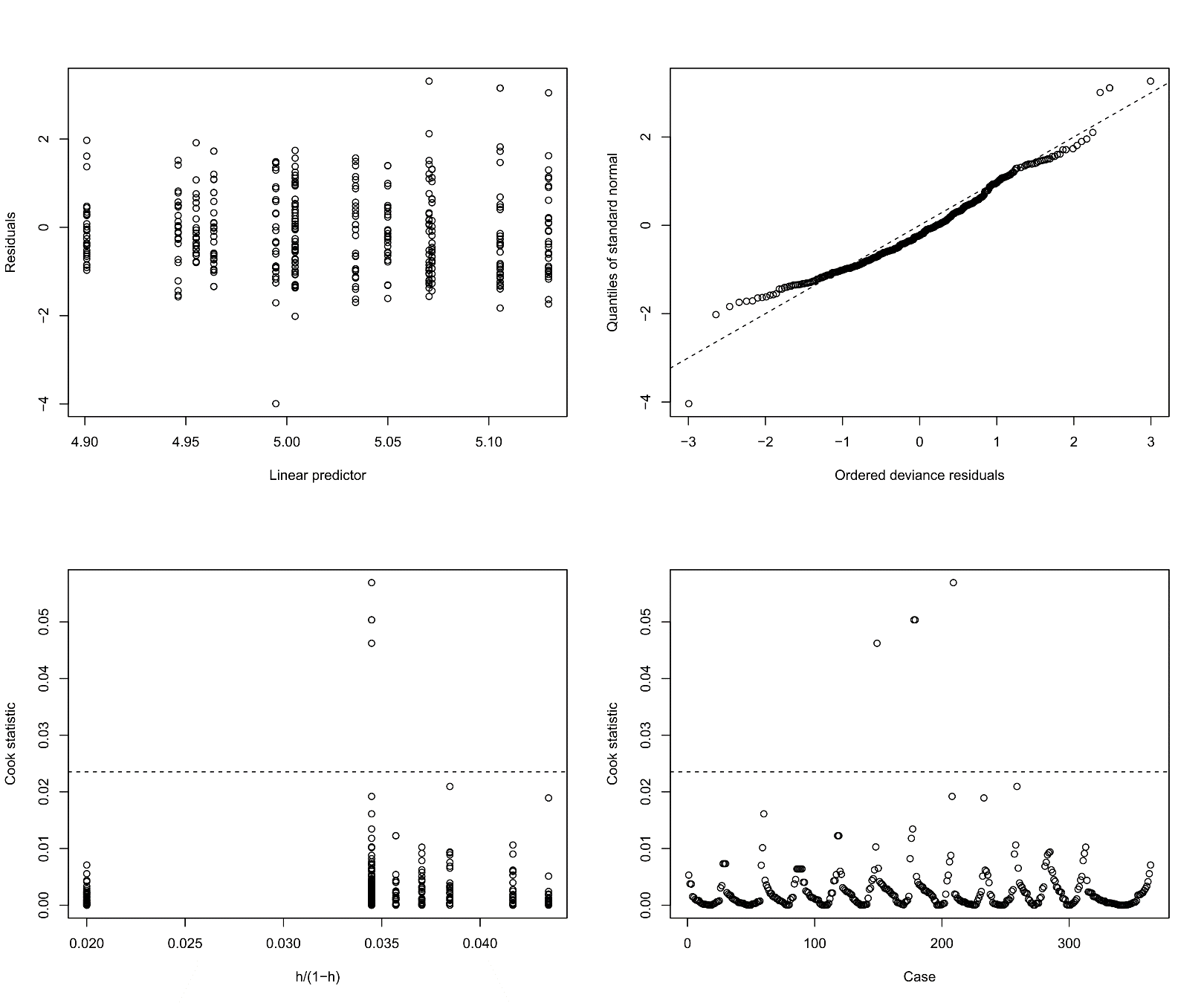


Figure S8: Diagnostic plots Gamma-GLM cold resistance in wild populations of the Queensland fruit fly.


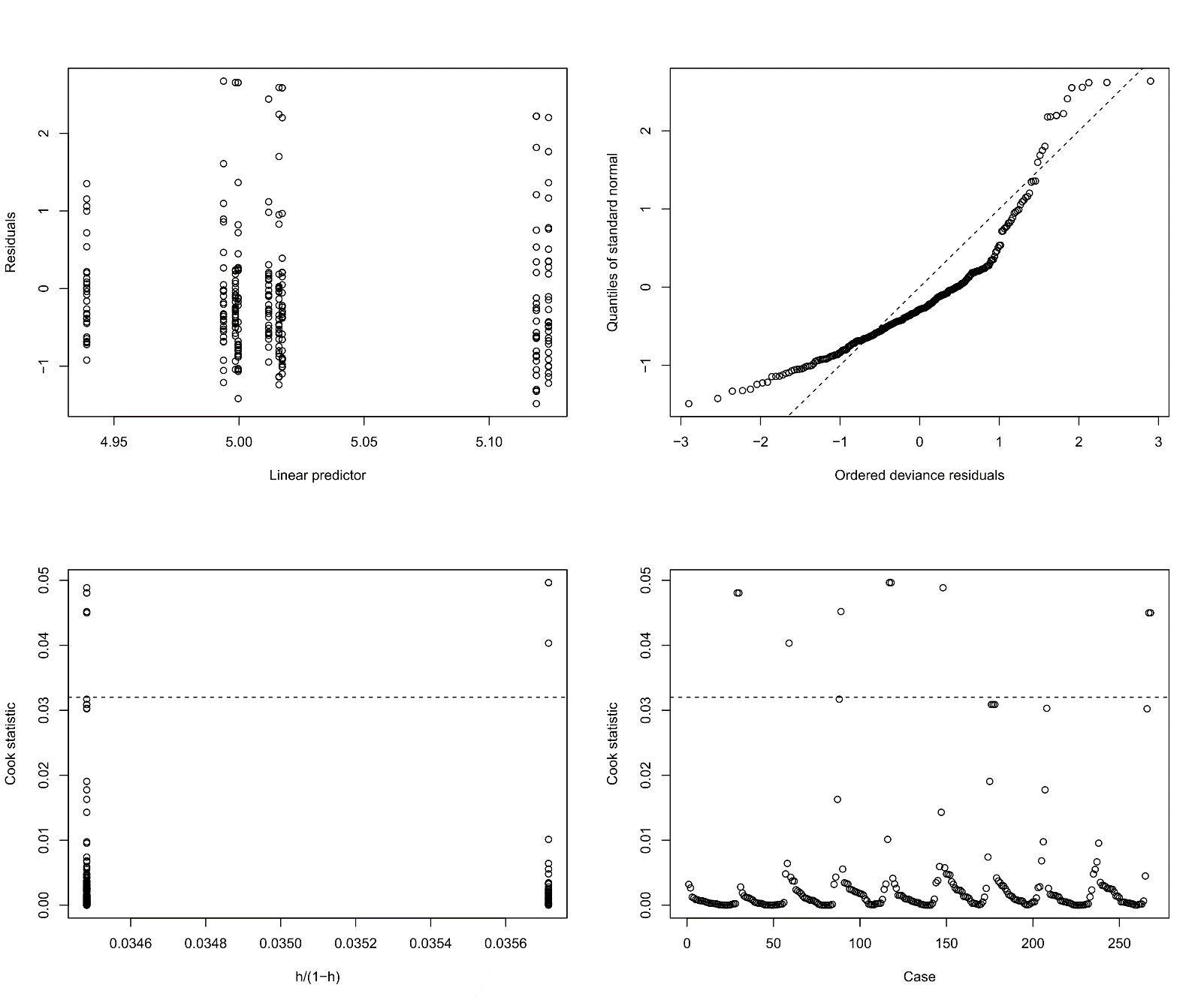


Figure S9: Diagnostic plots Gamma-GLM cold resistance in domesticated populations of the Queensland fruit fly.


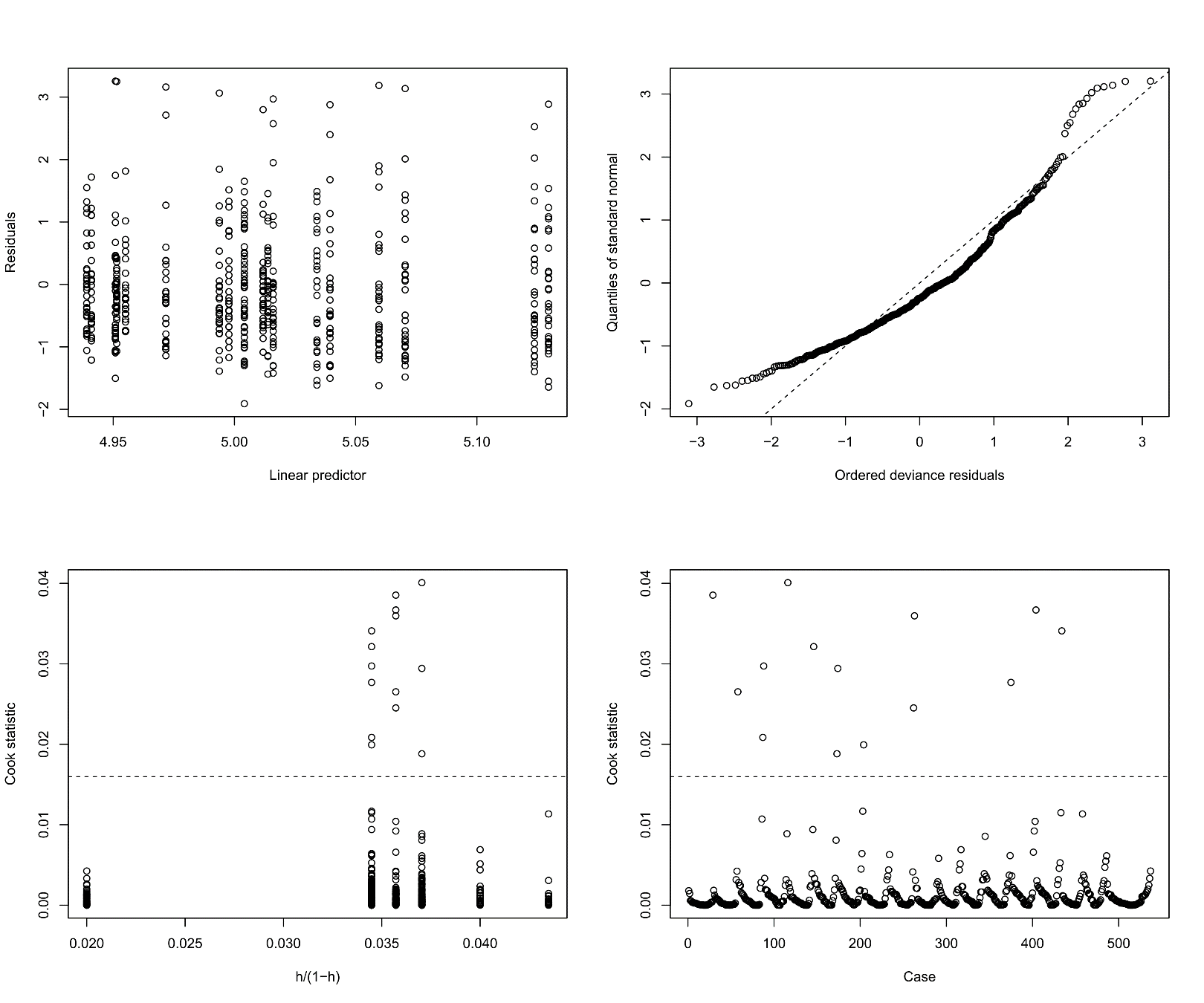


Figure S10: Diagnostic plots Gamma-GLM cold resistance change during domestication.


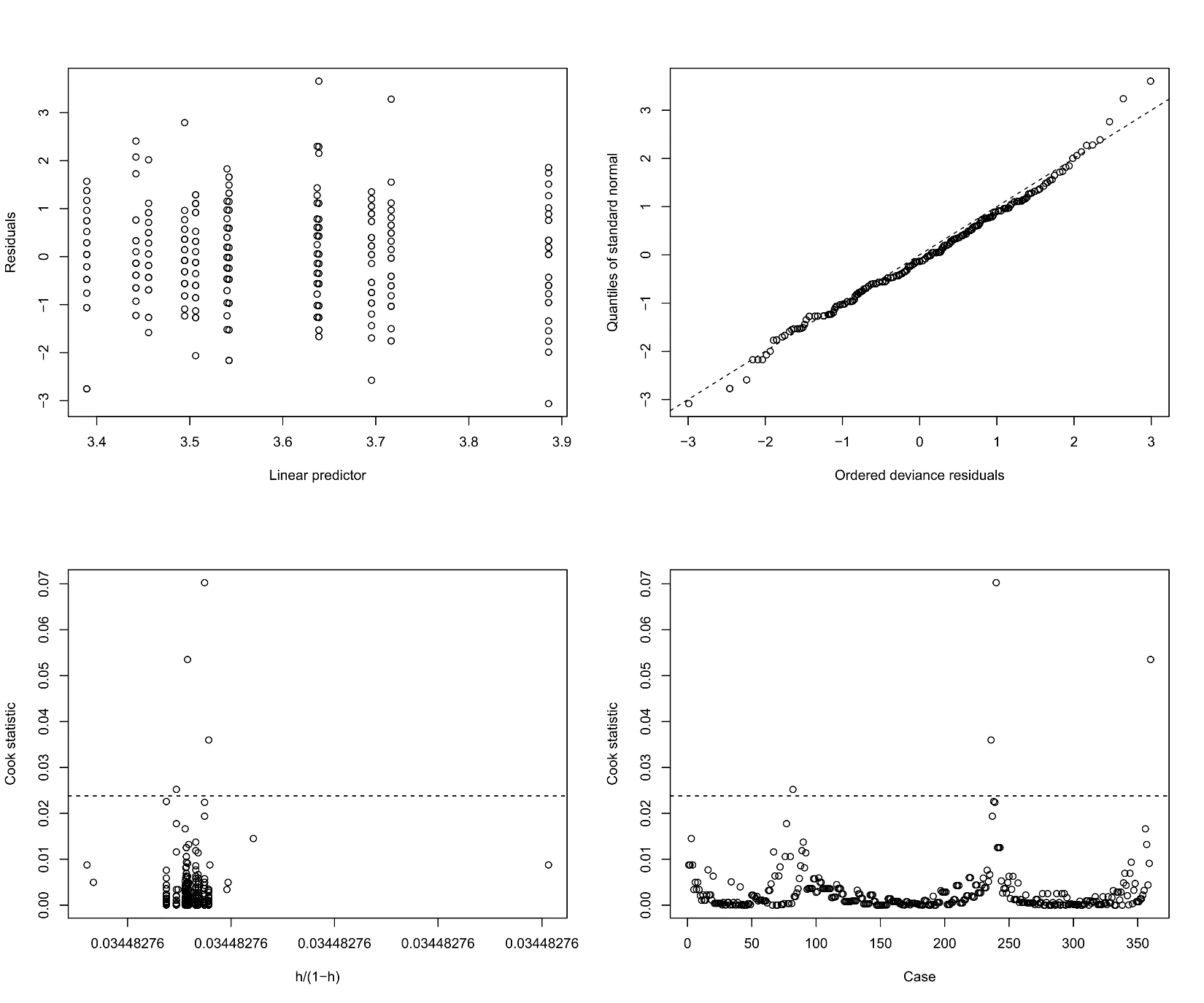


Figure S11: Diagnostic plots Gamma-GLM desiccation resistance in wild Qfly populations.


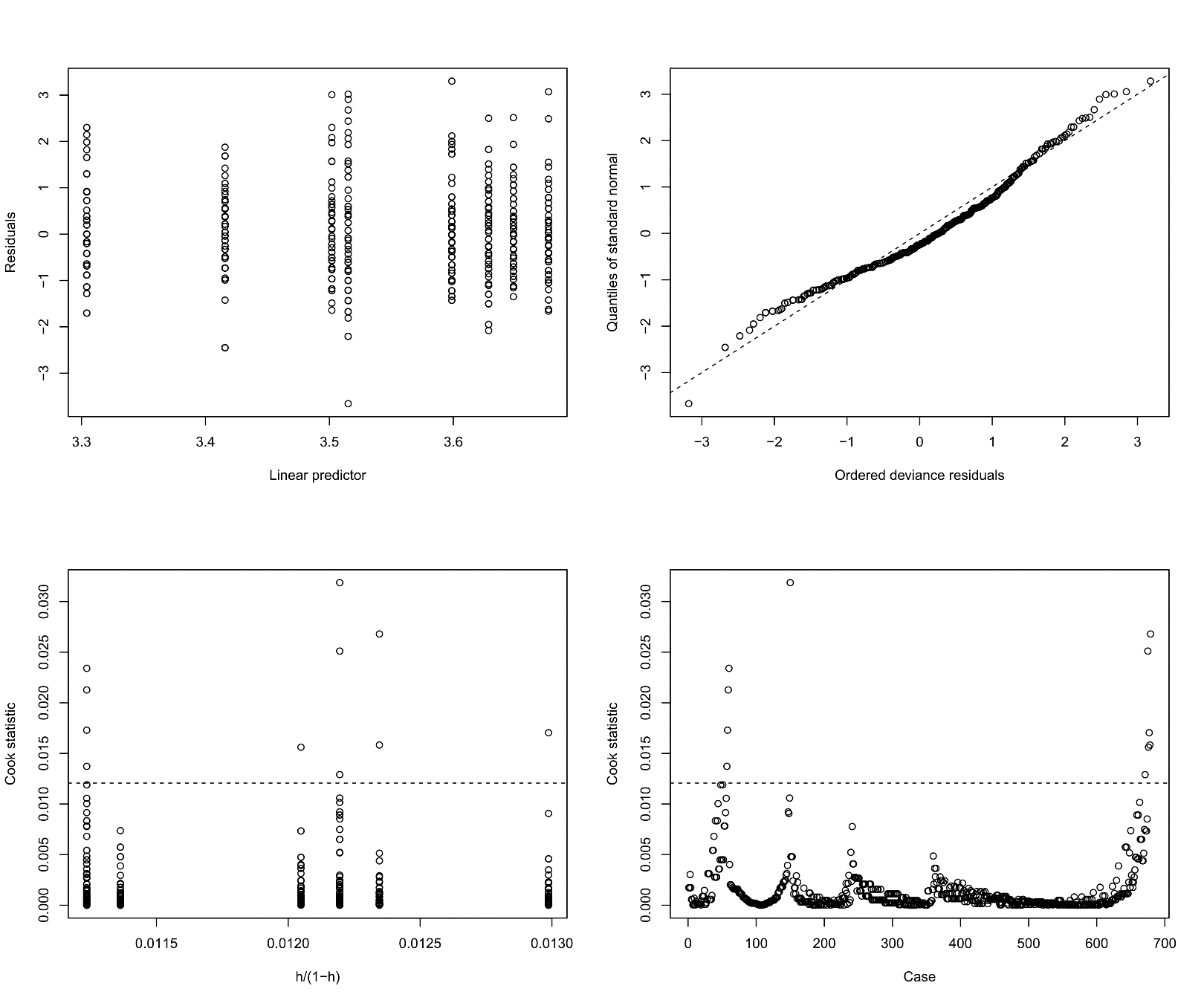


Figure S12: Diagnostic plots Gamma-GLM desiccation resistance in domesticated populations of the Queensland fruit fly.


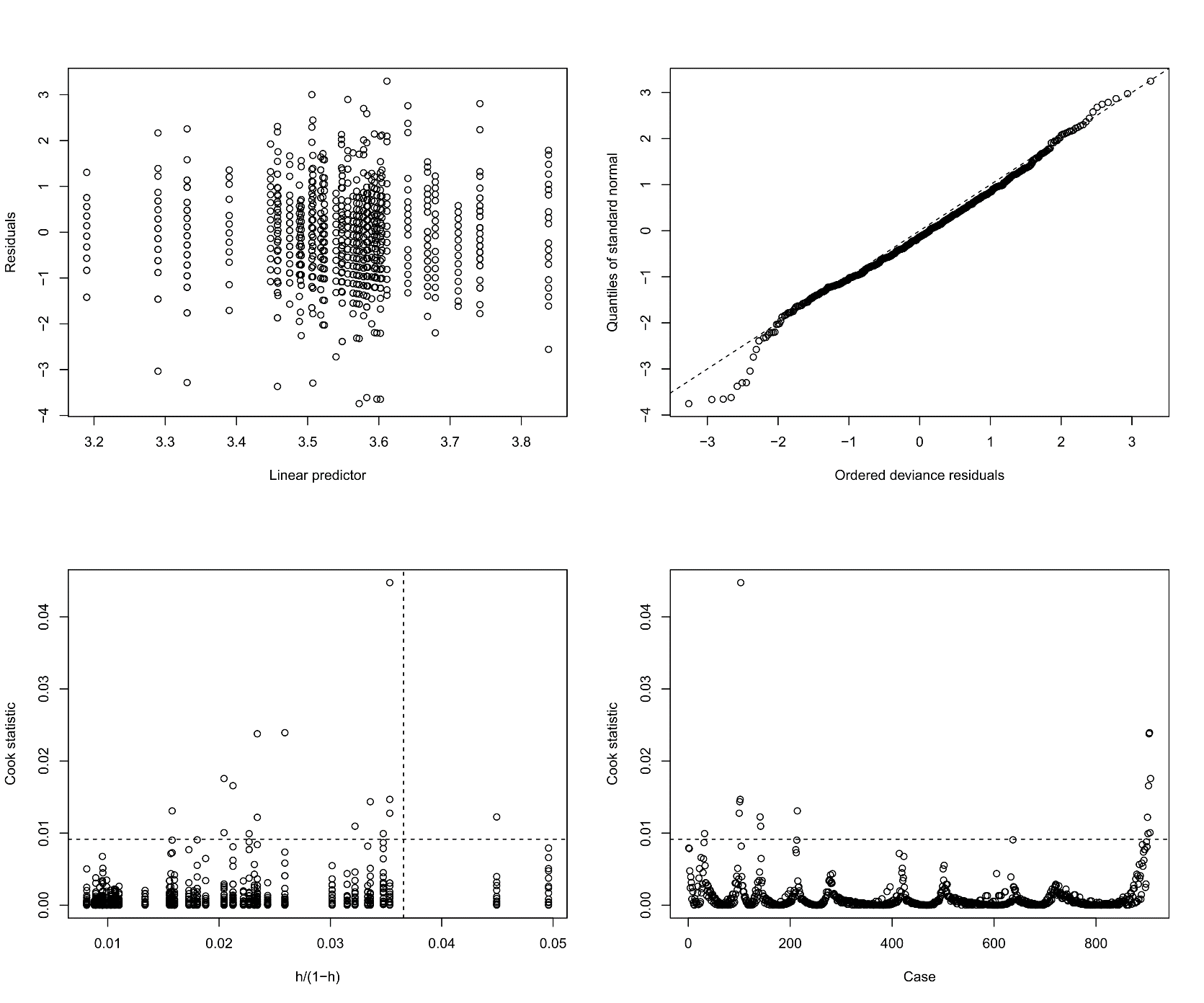


Figure S13: Diagnostic plots Gamma-GLM desiccation resistance change during domestication.


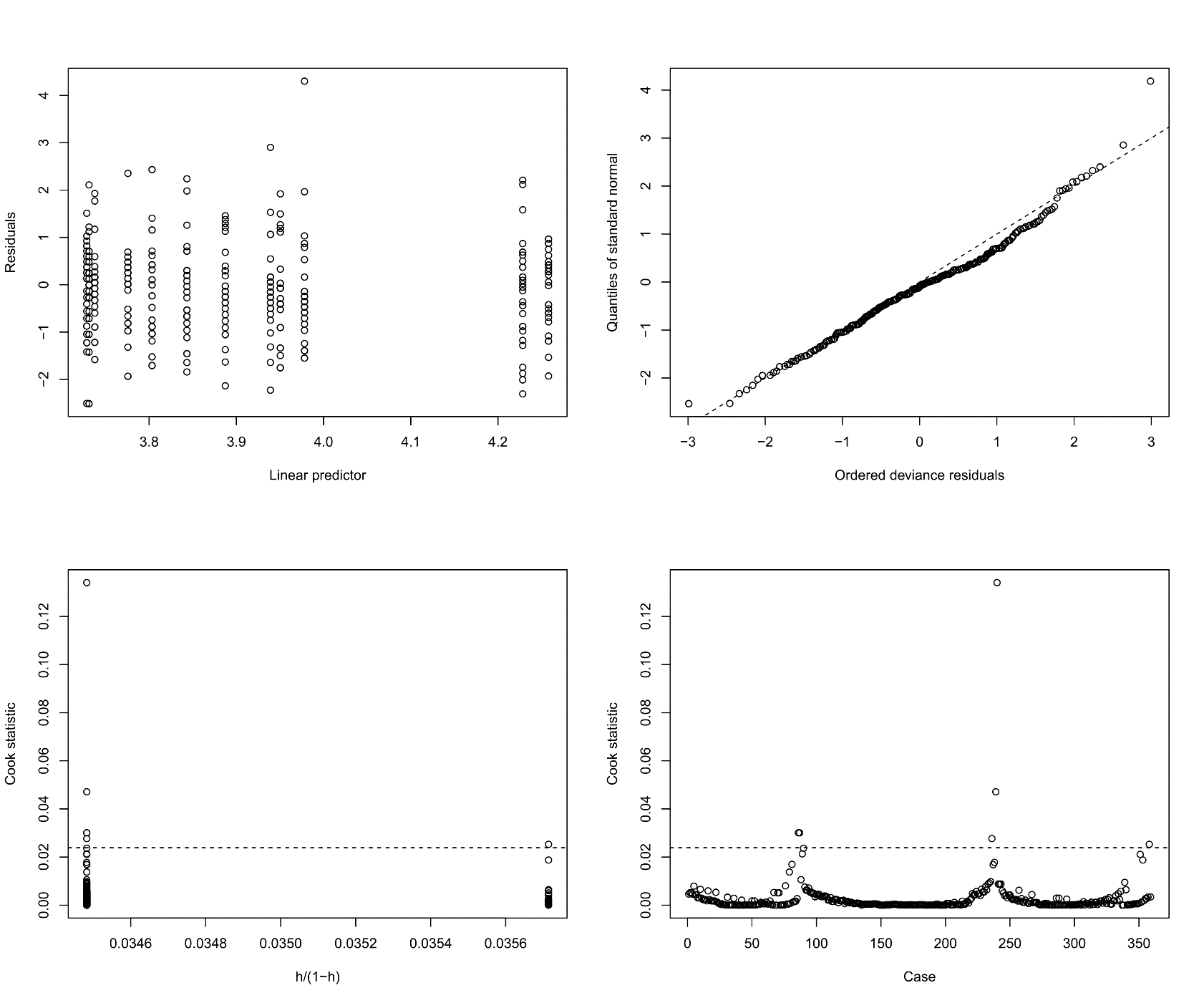


Figure S14: Diagnostic plots Gamma-GLM starvation resistance in wild Qfly populations.


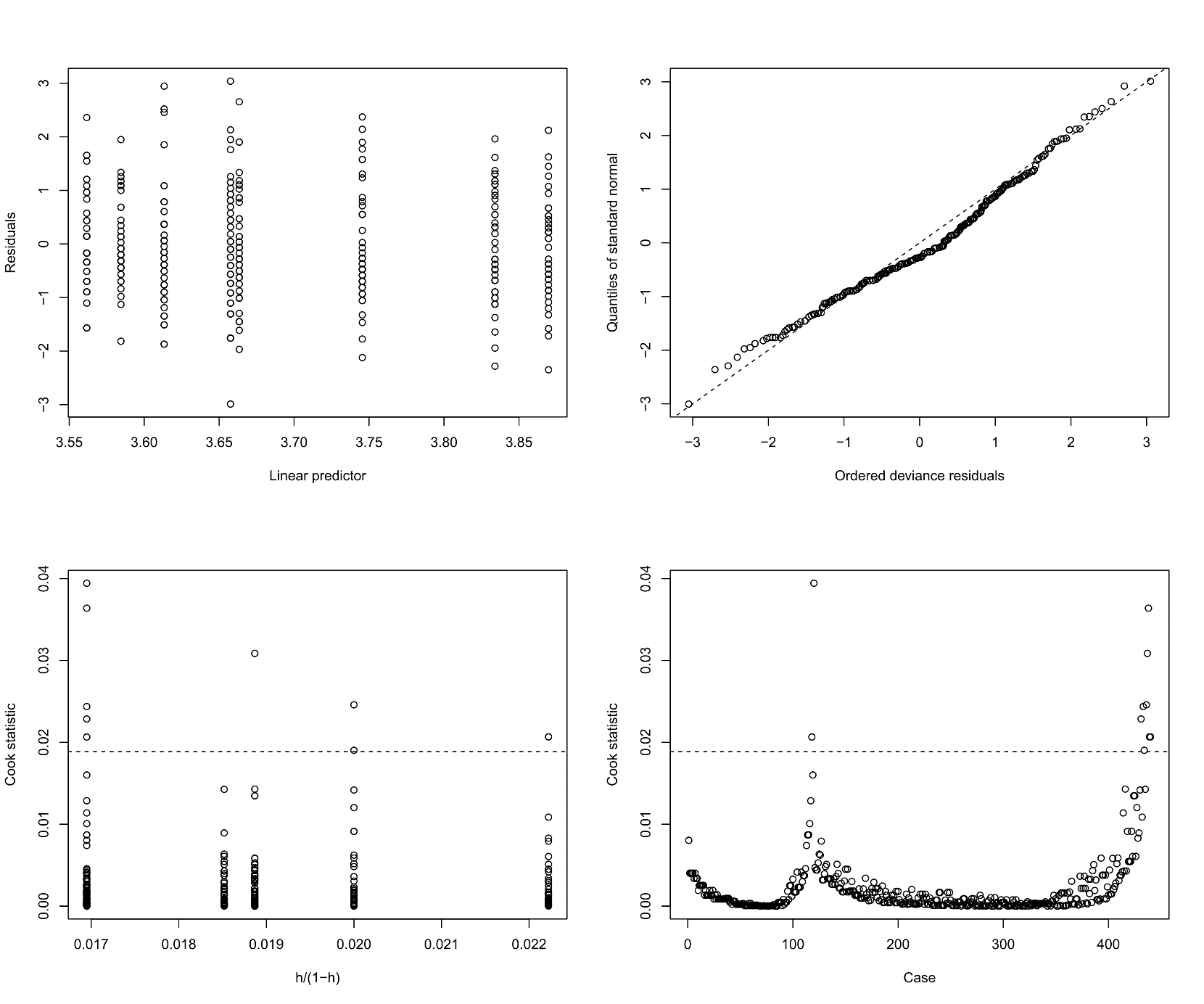


Figure S15: Diagnostic plots Gamma-GLM starvation resistance in domesticated populations of the Queensland fruit fly.


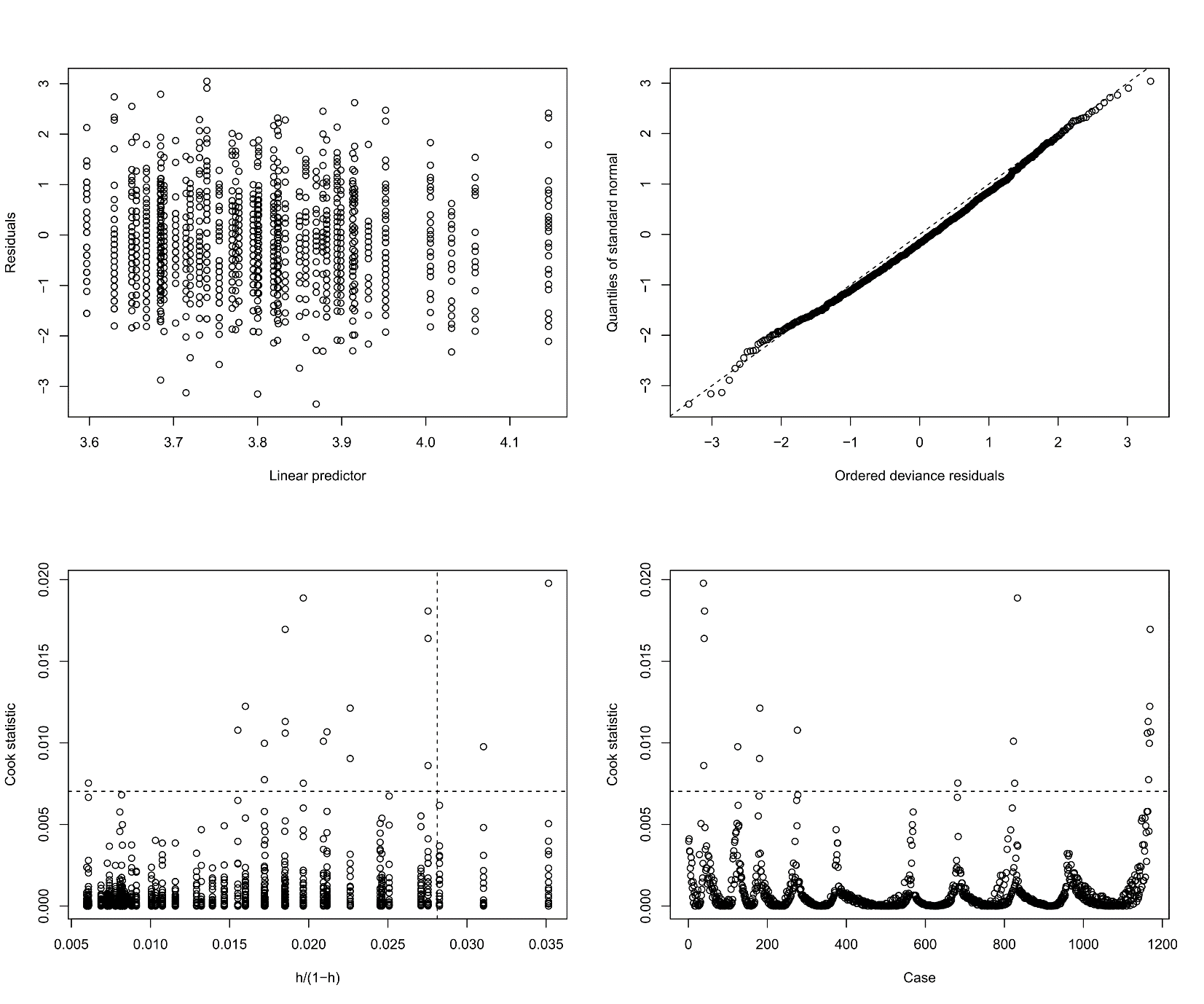


Figure S16: Diagnostic plots Gamma-GLM starvation resistance change during domestication.

**R packages used in the statistical analyses. R version 3.6.1 (2019-07-05)**

**Platform:** x86_64-w64-mingw32/x64 (64-bit)

**attached base packages:** *grid*, *stats*, *graphics*, *grDevices*, *utils*, *datasets*, *methods* and *base*

**other attached packages:** *raster(v.2.9-5)*, *rgdal(v.1.4-4)*, *ggrepel(v.0.8.1)*, *pander(v.0.6.3)*, *Perfor- manceAnalytics(v.1.5.3)*, *xts(v.0.11-2)*, *zoo(v.1.8-6)*, *vegan(v.2.5-5)*, *permute(v.0.9-5)*, *emmeans(v.1.3.5)*,

*boot(v.1.3-22)*, *nortest(v.1.0-4)*, *jtrans(v.0.2.1)*, *Hmisc(v.4.2-0)*, *Formula(v.1.2-3)*, *survival(v.2.44-1.1)*,

*lattice(v.0.20-38)*, *xtable(v.1.8-4)*, *psych(v.1.8.12)*, *egg(v.0.4.2)*, *gridExtra(v.2.3)*, *sp(v.1.3-1)*, *cowplot(v.1.0.0)*,

*ggpubr(v.0.2)*, *magrittr(v.1.5)*, *ggridges(v.0.5.1)*, *forcats(v.0.4.0)*, *stringr(v.1.4.0)*, *dplyr(v.0.8.3)*, *purrr(v.0.3.3)*,

*readr(v.1.3.1)*, *tidyr(v.1.0.0)*, *tibble(v.2.1.3)*, *ggplot2(v.3.2.1)*, *tidyverse(v.1.2.1)* and *wesanderson(v.0.3.6)*

**loaded via a namespace (and not attached):** *TH.data(v.1.0-10)*, *colorspace(v.1.4-1)*,

*ggsignif(v.0.5.0)*, *estimability(v.1.3)*, *htmlTable(v.1.13.1)*, *base64enc(v.0.1-3)*, *rstudioapi(v.0.10)*,

*mvtnorm(v.1.0-10)*, *lu- bridate(v.1.7.4)*, *xml2(v.1.2.0)*, *codetools(v.0.2-16)*, *splines(v.3.6.1)*, *mnormt(v.1.5-5)*,

*knitr(v.1.23)*, *zeallot(v.0.1.0)*, *jsonlite(v.1.6)*, *broom(v.0.5.2)*, *cluster(v.2.1.0)*, *compiler(v.3.6.1)*, *httr(v.1.4.0)*,

*back-ports(v.1.1.5)*, *assertthat(v.0.2.1)*, *Matrix(v.1.2-17)*, *lazyeval(v.0.2.2)*, *cli(v.1.1.0)*, *acepack(v.1.4.1)*, *html-*

*tools(v.0.3.6)*, *tools(v.3.6.1)*, *coda(v.0.19-2)*, *gtable(v.0.3.0)*, *glue(v.1.3.1)*, *reshape2(v.1.4.3)*, *Rcpp(v.1.0.3)*,

*cellranger(v.1.1.0)*, *vctrs(v.0.2.0)*, *nlme(v.3.1-140)*, *xfun(v.0.7)*, *rvest(v.0.3.4)*, *lifecycle(v.0.1.0)*, *MASS(v.7.3-*

*51.4)*, *scales(v.1.0.0)*, *hms(v.0.4.2)*, *parallel(v.3.6.1)*, *sandwich(v.2.5-1)*, *RColorBrewer(v.1.1-2)*, *yaml(v.2.2.0)*,

*rpart(v.4.1-15)*, *latticeExtra(v.0.6-28)*, *stringi(v.1.4.3)*, *checkmate(v.1.9.3)*, *rlang(v.0.4.2)*, *pkgcon-*

*fig(v.2.0.3)*, *evaluate(v.0.14)*, *htmlwidgets(v.1.3)*, *labeling(v.0.3)*, *tidyselect(v.0.2.5)*, *plyr(v.1.8.4)*, *R6(v.2.4.1)*,

*generics(v.0.0.2)*, *multcomp(v.1.4-10)*, *pillar(v.1.4.2)*, *haven(v.2.1.0)*, *foreign(v.0.8-72)*, *withr(v.2.1.2)*,

*mgcv(v.1.8-28)*, *nnet(v.7.3-12)*, *modelr(v.0.1.4)*, *crayon(v.1.3.4)*, *rmarkdown(v.1.16)*, *readxl(v.1.3.1)*,

*data.table(v.1.12.2)*, *digest(v.0.6.23)*, *webshot(v.0.5.1)*, *munsell(v.0.5.0)*, *viridisLite(v.0.3.0)*, *kableEx- tra(v.1.1.0)*

and *quadprog(v.1.5-7)*
